# Antagonistic transcriptome profile reveals potential mechanisms of action on *Xanthomonas oryzae* pv. *oryzicola* by the cell-free supernatants of *Bacillus velezensis* 504, a versatile plant probiotic bacterium

**DOI:** 10.3389/fcimb.2023.1175446

**Published:** 2023-05-30

**Authors:** Qi Zhou, Min Tu, Xue Fu, Ying Chen, Muyuan Wang, Yuan Fang, Yichao Yan, Guanyun Cheng, Yikun Zhang, Zhongfeng Zhu, Ke Yin, Youlun Xiao, Lifang Zou, Gongyou Chen

**Affiliations:** ^1^ Shanghai Collaborative Innovation Center of Agri-Seeds/School of Agriculture and Biology, Shanghai Jiao Tong University, Shanghai, China; ^2^ Rubber Research Institute, Chinese Academy of Tropical Agricultural Sciences, Haikou, China; ^3^ College of Plant Protection, Yunnan Agricultural University, Kunming, China; ^4^ Institute of Plant Protection, Hunan Academy of Agricultural Sciences, Changsha, China; ^5^ Key Laboratory of Microbial Metabolism, School of Life Sciences and Biotechnology, Shanghai Jiao Tong University, Shanghai, China

**Keywords:** transcriptome profiling, antagonism mechanism, biocontrol agent, *Bacillus velezensis*, *Xanthomonas oryzae* pv. *oryzicola*

## Abstract

Bacterial leaf streak (BLS) of rice is a severe disease caused by the bacterial pathogen *Xanthomonas oryzae* pv. *oryzicola* (*Xoc*) that has gradually become the fourth major disease on rice in some rice-growing regions in southern China. Previously, we isolated a *Bacillus velezensis* strain 504 that exhibited apparent antagonistic activity against the *Xoc* wild-type strain RS105, and found that *B. velezensis* 504 was a potential biocontrol agent for BLS. However, the underlying mechanisms of antagonism and biocontrol are not completely understood. Here we mine the genomic data of *B*. *velezensis* 504, and the comparative transcriptomic data of *Xoc* RS105 treated by the cell-free supernatants (CFSs) of *B*. *velezensis* 504 to define differentially expressed genes (DEGs). We show that *B*. *velezensis* 504 shares over 89% con*s*erved genes with FZB42 and SQR9, two representative model strains of *B*. *velezensis*, but 504 is more closely related to FZB42 than SQR9, as well as *B*. *velezensis* 504 possesses the secondary metabolite gene clusters encoding the essential anti-*Xoc* agents difficidin and bacilysin. We conclude that approximately 77% of *Xoc* RS105 coding sequences are differentially expressed by the CFSs of *B*. *velezensis* 504, which significantly downregulates genes involved in signal transduction, oxidative phosphorylation, transmembrane transport, cell motility, cell division, DNA translation, and five physiological metabolisms, as well as depresses an additional set of virulence-associated genes encoding the type III secretion, type II secretion system, type VI secretion system, type IV pilus, lipopolysaccharides and exopolysaccharides. We also show that *B*. *velezensis* 504 is a potential biocontrol agent for bacterial blight of rice exhibiting relative control efficiencies over 70% on two susceptible cultivars, and can efficiently antagonize against some important plant pathogenic fungi including *Colletotrichum siamense* and *C*. *australisinense* that are thought to be the two dominant pathogenic species causing leaf anthracnose of rubber tree in Hainan province of China. *B*. *velezensis* 504 also harbors some characteristics of plant growth-promoting rhizobacterium such as secreting protease and siderophore, and stimulating plant growth. This study reveals the potential biocontrol mechanisms of *B*. *velezensis* against BLS, and also suggests that *B*. *velezensis* 504 is a versatile plant probiotic bacterium.

## 1 Introduction

Rice is an important worldwide staple crop, however, stable rice production is threatened by various diseases, including fungal and bacterial diseases. Bacterial blight (BB) of rice caused by *Xanthomonas oryzae* pv. *oryzae* (*Xoo*), and bacterial leaf streak (BLS) of rice caused by *X*. *oryzae* pv. *oryzicola* (*Xoc*), are important bacterial diseases in rice growing areas of Asia (Nino-Liu et al., 2006). These two diseases often result in rice yield reduction by 10-30% especially in the south of China (Li et al., 2019). Currently, the main control measures for these two bacterial diseases include the development of resistant varieties of rice and the application of chemical bactericides (Xu et al., 2015). The interaction between *Xoc* and rice does not conform to the gene-for-gene hypothesis (Li et al., 2019). Some rice varieties containing *xa5*, *Xa23*, *Xa21* and other resistance genes showed better resistance to *Xoo*, but exhibited susceptibility to *Xoc* (Liu et al., 2021b). Many hybrid rice varieties in China are susceptible to *Xoc*, and some of which were highly susceptible (Li et al., 2019). At present, the control of BLS mainly depends on chemical agents, such as zinc thiazole or cupric bactericides (Yang et al., 2021). The use of these bactericides is not only easy to cause phytotoxicity and make pathogens to develop resistance, but also damages the ecological environment. Therefore, biological control measures have been strongly promoted, and the study of screening antagonistic microorganisms from agricultural ecological environment to control BB and BLS has become an important research direction (Yang et al., 2023).

*Pseudomonas* spp. and *Bacillus* spp. are used commercially as biocontrol agents against plant diseases in agricultural production. Some studies have shown that some species of Pseudomonads such as *P*. *entomophila*, *P*. *putida* group members *P*. *soli* and *P*. *mosselii*, and *P*. *oryziphila*, a novel species recently identified in our previous study, exhibited antagonistic activity against *Xoo* and *Xoc* (Li et al., 2013; Pascual et al., 2014; Yang et al., 2021). *Bacillus* spp. is the most commercially used biocontrol bacteria because of its good stress resistance, producing many kinds of secondary metabolites, and having the functions of disease prevention, growth promotion and environmental restoration (Baard et al., 2023; Poulaki and Tjamos, 2023). At present, *B*. *amyloliquefaciens* and *B*. *velezensis* are successful cases of *Bacillus* spp. used for biological control of BB and BLS. *B*. *amyloliquefaciens* Lx-11 is the first commercial biopesticide created by *B*. *amyloliquefaciens* in China (Zhang et al., 2012). It was registered for the control of BB and BLS, the control efficacy ranging from 60.2% to 70.6% (Zhang et al., 2012). *B*. *velezensis* FZB42 is a model strain of plant growth-promoting rhizobacterium (PGPR) for stimulation of induced systemic resistance, and suppression of plant pathogens such as fungi, bacteria (*Xoo* and *Xoc*), oomycetes, and nematodes (Wu et al., 2015; Fan et al., 2018; Fan et al., 2019; Han et al., 2021; Mácha et al., 2021). Previously, we identified 16 species of *Bacillus* including *B*. *cereus*, *B*. *altitudinis* and *B*. *velezensis* with antagonistic effects against *Xoc* in a biocontrol bacterial pool of 233 strains that were isolated from the 248 rhizosphere soil samples collected from 23 provinces in China (Li et al., 2019; Li et al., 2020a; Huang et al., 2021). This suggests that *Bacillus* is a resource reservoir to be excavated for the BLS control.

The present taxon of *B*. *velezensis* is an integrated taxon that includes all the strains previously classified as *B*. *velezensis*, *B*. *methylotrophicus* and *B*. *amyloliquefaciens* subsp. *plantarum* (Dunlap et al., 2015; Dunlap et al., 2016). It is predicted that about 10% of the total genome sequence of the model strain FZB42 encodes at least 13 gene clusters that devoted to synthesizing active secondary metabolites, which play effective roles in the controls of fungal, bacterial, viral and nematode diseases (Rabbee et al., 2019). These bioactive secondary metabolites have been identified in four types of cyclic lipopeptides (such as surfactin, bacillomycin-D, fengycin, and bacillibactin, an iron-siderophore), three polyketones (such as macrolactin, bacillaene and difficidin), one dipeptide antibiotic (bacilysin), two bacteriocins (plantazolicin and amylocyclicin), and volatile substances (acetoin and 2, 3-butandiol) (Chowdhury et al., 2015; Fan et al., 2018; Rabbee et al., 2019). Difficidin and bacilysin are major antibacterial agents produced by FZB42 to exert biocontrol activity of the BB and BLS caused by *Xoo* and *Xoc*, respectively (Wu et al., 2015). However, the mechanisms by which the active compounds produced by *B*. *velezensis* strains affect *Xoc* pathogenicity are little understood. In recent years, with the adoption of different antagonistic target pathogens, new isolates of *B*. *velezensis*, such as J17-4, NKMV-3 and HNA3, have been continuously isolated and identified (Shi et al., 2022; Vignesh et al., 2022; Zaid et al., 2022). Some new biocontrol potentials of *B*. *velezensis* strains are being explored.

Our laboratory has been working to develop biological control methods to control BLS. Previously, we isolated and identified a *B*. *velezensis* strain 504 that exhibited apparently antagonistic activity against the *Xoc* wild-type strain RS105 (Li et al., 2019). In this study, we completed the whole genome sequencing and conducted the comparative genomic analysis of *B*. *velezensis* 504 with its closely related *B*. *velezensis* strains. In addition, we compared the transcriptome profiling of *Xoc* RS105 treated by the cell-free supernatants (CFSs) of *B*. *velezensis* 504 and revealed the potential mechanisms of action of *B*. *velezensis* 504 on *Xoc*. We also analyzed the antagonistic spectrum of *B*. *velezensis* 504 against phytopathogenic fungi such as the pathogens of rice blast, gray mold of vegetables and leaf anthracnose of rubber tree. These studies suggest that *B*. *velezensis* 504 is a versatile plant probiotic bacterium.

## 2 Materials and methods

### 2.1 Bacteria, pathogens, and plant materials used in this study

*B. velezensis* 504 was isolated from the rhizosphere soil samples of spinach in Xiyang Village, Sanming City, Fujian province, China, cultured in Luria-Bertani (LB) medium at 28°C.

All *Xanthomonas* strains were cultured in nutrient agar (NA) or nutrient broth (NB) medium at 28°C. All fungal strains were cultured in Potato Dextrose Agar (PDA) medium at 28°C (Yang et al., 2023). Rice seeds Yuanfengzao, were provided by Dr. Youlun Xiao from Institute of Plant Protection, Hunan Academy of Agricultural Sciences. Rice seeds IR24 and Nipponbare used in this study were from our laboratory.

### 2.2 Antimicrobial activity assays

The Kirschbaum-Bauer (KB) method was used to evaluate the antagonism of *B*. *velezensis* strain 504 against *Xoc* RS105, *Xoo* PXO99^A^, and phytopathogenic fungi, including *Fusarium graminearum* causing fusarium head blight, *Botrytis cinerea* causing gray mold disease of vegetables, *Magnaporthe oryzae* causing rice blast, *F*. *oxysporum* causing root rot disease of tomato, *Colletotrichum siamense* and *C*. *australisinense* causing leaf anthracnose of rubber tree (Liu et al., 2018; Liu et al., 2023). All isolates were tested in triplicate, and inhibition zones against *Xanthomonas* were measured 2-3 days after co-cultivation at 28°C on nutrient agar (NA), and inhibition zones against fungi were measured 5-7 days after co-cultivation at 25°C. The antifungal activity of *B*. *velezensis* 504 was further studied by the following formula:


$$\begin{array}{c} \text{Antagonistic}\;\text{activity}(\%)\\ =\text{semidiameter}\;\text{in}\;\text{treatment}\times 2/(\text{semidiameter}\;\text{in}\;\text{control}/2)\\ \times 100\% \end{array}$$

### 2.3 Plant growth-promoting characteristics analysis of *B*. *velezensis* 504

The biological functional studies of *B*. *velezensis* 504 were performed as previously reported studies (Tang et al., 2023). We detected the siderophore production from *B*. *velezensis* 504 by the assay of chrome azurol (CAS) agar plate (Milagres et al., 1999). To test the protease activity of *B*. *velezensis* 504, 1 μl bacterial solution was added to protease assay plates and incubated at 28°C for 3 days. Then, the plates were observed for the formation of transparent circles due to the decomposition of substrates. Drop 1 μl of cultured solution of *B*. *velezensis* 504 on the cellulase activity-tested medium, inorganic phosphorus culture plates, and inorganic potassium culture plates and cultured at 28°C for 4, 7, 7 days, respectively. Each inoculation of plates was conducted in three replications.

### 2.4 Genomic analysis and phylogenomic for *B*. *velezensis* 504

The whole genome of *B*. *velezensis* 504 was sequenced using the Pacific Biosciences platform and the Illumina Miseq platform at Personalbio (Shanghai, China). The complete genome sequences of *B*. *velezensis* 504 were deposited in GenBank under accession number, CP092439 for chromosome, and CP092440 for plasmid. After obtaining the sequence, we used various databases to add annotations of function genes. CAZy (Carbohydrate-Active enzymes) (Hmmscan, v3.1b2, February 2015) was used to predict the presence of carbohydrate active enzyme genes in the genome sequence; COG (cluster of orthologous group) (eggNOG-mapper, v4.5, November 2017) and GO (Gene Ontology) (Interpro, v66.0, November 2017) were used to annotate protein-coding genes in the whole genome; KEGG (Kyoto Encyclopedia of Genes and Genomes) (KAAS, v2.1) was used to annotate its metabolic pathway. The average nucleotide identity (ANI) values among 12 genome sequences *B*. *velezensis* 504 and other *Bacillus* strains, including *B*. *velezensis* FZB42, *B*. *velezensis* SQR9, *B*. *siamensis* KCTC 13613, *B*. *amyloliquefaciens* DSM 7, *B*. *subtilis* subsp. *subtilis* str. 168, *B*. *halotolerans* ZB201702, *B*. *subtilis subsp*. *spizizenii* W23, *B*. *atrophaeus* subsp. *globigii* BSS, *B*. *licheniformis* SCDB 14, *B*. *mojavensis* UCMB5075, and *B*. *vallismortis* Bac111 were calculated using the J Species WS Online Service (Richter et al., 2016). We obtained and compared general characteristic information for the three strains in NCBI (http://www.ncbi.nlm.nih.gov/).

Pan-genomic analysis of the *B*. *velezensis* genomes was conducted using the BPGA v1.3.0 tool (Chaudhari et al., 2016). For the extraction of the core-genome the default values of the tool were employed, i.e., USEARCH software v10.0.240 with a threshold of sequence identity equal to 0.5 or 50% (Yoon et al., 2017). The MUSCLE v3.8.31 software was used to align the concatenated amino acids of the core-genome. The pan-genomic analysis was repeated in the *B*. *velezensis* genomes, including the genomes of *B. velezensis* 504, *B. velezensis* FZB42 and *B. velezensis* SQR9. We also used KEGG and COG to annotate these unique, accessory and core genes. Finally, we drew a Venn diagram through TBtools software.

Collinearity of the conserved and highly orthologous genomic regions were determined and plotted among *B. velezensis* 504, *B. velezensis* FZB42 and *B. velezensis* SQR9 by using Mauve software v2.4.0 with default parameters (Darling et al., 2004). The colored, locally collinear blocks (LCBs) show the conserved and highly similar genomic regions. Each continuously shaded area is an LCB. The colored lines between the two genomes trace the LCBs of each homologous. We selected rhizosphere colonization-related genes among *B. velezensis* 504, *B. velezensis* FZB42 and *B. velezensis* SQR9 for genetic similarity comparison by using NovoPro (https://www.novopro.cn/tools/ident_sim.html).

### 2.5 The antiSMASH analysis

The genomes of *B. velezensis* 504, *B. velezensis* FZB42 and *B. velezensis* SQR9 were analyzed by antiSMASH 5.0 with website (https://antismash.secondary-metabolites.org) to predict the putative secondary metabolite biosynthesis gene clusters (Blin et al., 2019). Detailed gene cluster information was obtained from the GenBank databases.

### 2.6 Biocontrol assays

Biocontrol assays in rice fields were performed as previous research (Yang et al., 2023). Ten leaves from the highly susceptible rice cultivars Yuanfengzao, IR24 and Nipponbare rice at booting stage were inoculated with *Xoo* PXO99^A^ (OD_600 =_ 0.3) by spray inoculation. *B*. *velezensis* 504 cell-culture broth (CCB) preventive treatment (504-Pre) and treatment (504-Tre) indicated that spraying rice leaves with *B*. *velezensis* 504 CCB (OD_600 =_ 1.0) 12 hours prior to and after inoculation with PXO99^A^, respectively. Sterile water-treated rice cultivars were used as control (CK). BB disease of rice severity under all groups was investigated after 15 days. Relative spot area was calculated by the formula: Spot area= (spot area of this leave/total area of this leave) × 100%. The inhibitory percentages (IPs) were calculated by the formula: IP = (1-spot area of treatment/spot area of control) × 100%. Three independent experiments and three technical replicates were performed. Statistical analysis of results was determined by ANOVA test with significant difference (p< 0.05), and graphs were obtained with GraphPad Prism 9 version.

### 2.7 Growth promotion assays

To explore whether *B. velezensis* 504 present the ability to promoter plant growth of pak choi (*B*. *campestris* sp. *chinensis L.*), we measured growth parameters including dry weight of their aboveground parts. *B. velezensis* 504 suspension was diluted 10 and 50 times with sterile water and then irrigated to the soil prior to pakchoi sowing. Seeding with conventional method was carried out as control (CK). We randomly selected 30 plants in each treatment, and measured the dry weight of mature pakchoi cabbages after picking. Statistical analysis of results was determined by ANOVA test with significant difference (p< 0.05), and graphs were obtained with GraphPad Prism 9 version.

### 2.8 Preparation of biological samples used for RNA-seq

Cell-free supernatants (CFSs) were used to evaluate influence of *B. velezensis* 504 extracellular metabolites on the growth of *Xoc* RS105 as previous research with modifications (Jeong et al., 2017). *B. velezensis* 504 was shaking incubated in 250 ml NB medium at 28°C for overnight (adjusted OD_600_ to 2.0), centrifuged to remove bacterial cells and collected 200 ml supernatant as CFSs by 0.22um filtration. After freeze-drying, the powder of *B. velezensis* 504 CFSs were dissolved with sterile water (10 ml) for antagonistic activity determination. *In vitro* studies, 30, 40, 50, and 60 µL *B. velezensis* 504 CFSs (20 times concentration) was added with 2 ml RS105 inoculum (adjusted OD_600_ to 1.0) and incubated shaking at 28°C for overnight to determine the effective bactericidal concentration of *B. velezensis* 504 (**Supplementary Figure 4B**). The negative control consisted of sterile water and 2 ml RS105. For RNA-Seq analysis, *Xoc* RS105 that were centrifuged and transferred to liquid XOM3 medium (Yang et al., 2021), which is a nutrient-poor artificial medium mimicking the infection scenarios in the host rice, and can artificially induce *hrp* gene expression, (adjusted OD_600_ to 1.0) performed as negative controls. Twenty-fold concentrated *B. velezensis* 504 CFSs (50 µL) were added to *Xoc* RS105 that was re-suspended in XOM3 medium (adjusted OD_600_ to 1.0) and further incubated at 28°C shaking at 180 rpm for 6 h, 12 h, 24 h. Total RNAs of RS105 samples with three replicates from treatments (5046h1, 5046h2, 5046h3, 50412h1, 50412h2, 50412h3, 50424h1, 50424h2, and 50424h3) and controls (CK6h1, CK6h2, CK6h3, CK12h1, CK12h2, CK12h3, CK24h1, CK24h2, and CK24h3) grown on the tubes described at three time points were extracted for Illumina HiSeq sequencing. Finally, we sent all prepared samples above to Personalbio (Shanghai, China) for transcriptome sequencing analysis.

### 2.9 RNA sequencing, functional annotation and gene expression analysis

Based on the reference genome (https://www.ncbi.nlm.nih.gov/nuccore/CP011961.1), the company calculated the expression levels of each gene in each treatment group. The transcriptome samples were further analyzed for differential expression, enrichment, and clustering. Since we set up both control and treatment groups in the experiment, the comparison of gene expression levels between the two groups can reflect differential expression information. We used DESeq for differential analysis of gene expression, also used GO and KEGG to annotate function genes. Finally, we used Power Point software to draw a Venn diagram to show part of the results of the difference expression. Heatmaps were produced by TBtools software (Chen et al., 2020). Raw transcriptome data of the eighteen samples were deposited in the SRA database (Sequence Read Archive, NCBI) with the accession number PRJNA946931. Quantitative Real-time PCR (qRT-PCR) was employed with SYBR Green I Mix (TransGen, Beijing, China) and ABI 7500 software according to the manufacturer’s instructions. *gyrB* and *rpoD* were used as house-keeping genes for validation of fifteen differentially expressed genes in RS105. Specific primers were designed with Primer 3 online platform (**Supplementary Table 1**). The 2^−ΔΔCT^ method was used for relative quantification of gene expression. Spearman correlation test was conducted to measure the correlation between qRT-PCR (log2-expression fold change data) and RNA-Seq (log2-fold change) results using SPSS Version 20.0 and determine the reliability of RNA-Seq.

## 3 Results

### 3.1 Genome features and comparative genomics analysis of *B*. *velezensis* 504

**Figure 1:**
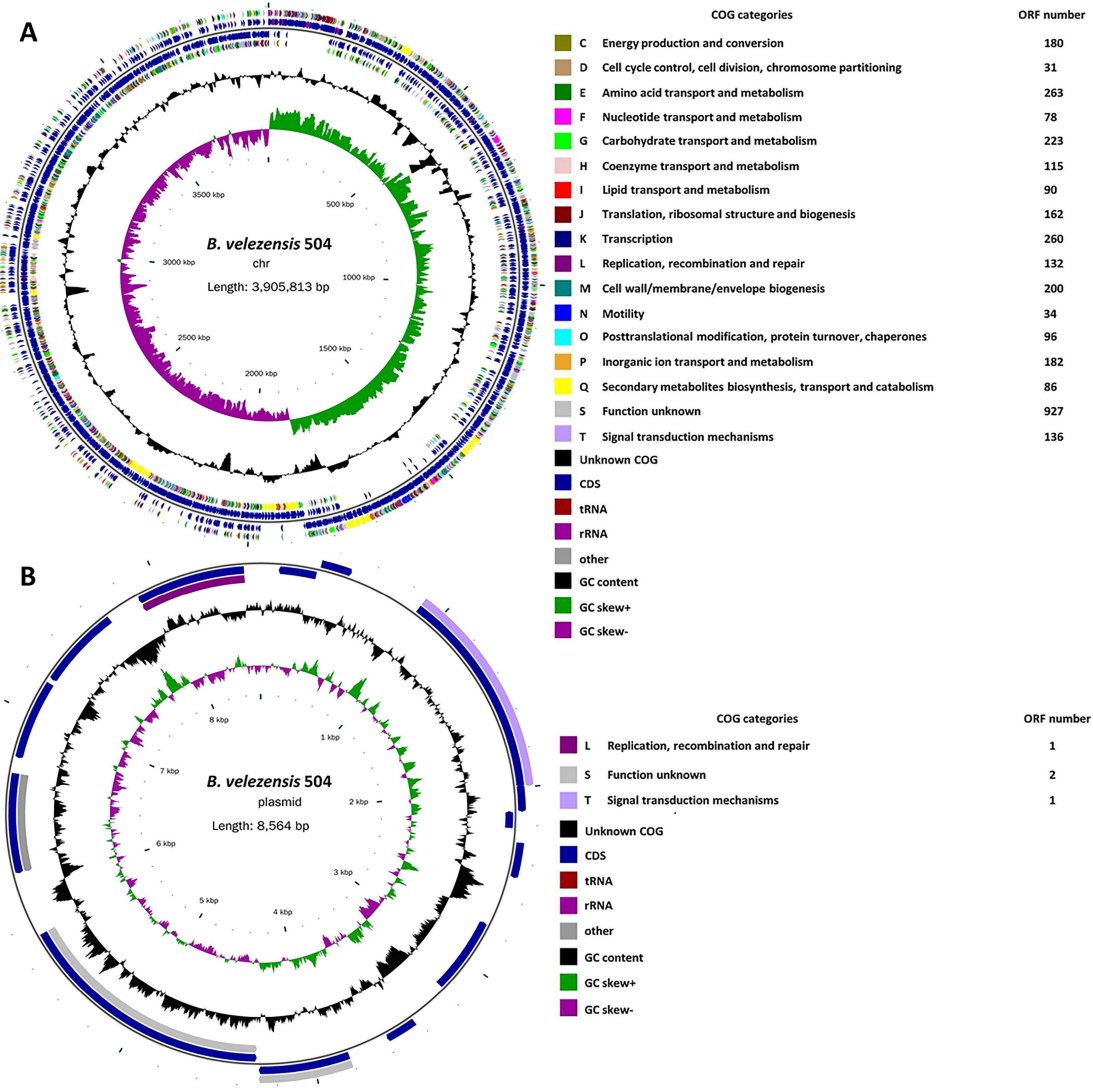
The complete genome map of *B. velezensis* 504 with its genomic features. **(A)** Circular map of *B. velezensis* 504 chromosome. The GC content of the strain 504 chromosome was 46.6% and contained 3,853 protein coding sequences (CDSs), 27 rRNA genes, 86 tRNA genes and 217 other non-coding RNA genes. **(B)** Circular map of *B. velezensis* 504 plasmid. The GC content of the 8,564 bp-plasmid was 39.86% and different from *B. velezensis* strains FZB42 and SQR9.

Previously, we isolated the bacterial strain 504, which displayed significant antagonistic activity against *Xoc*, from soil samples taken from the spinach rhizosphere at Xiyang Village of Sanming City in the province of Fujian, China, on February 21, 2018 (Li et al., 2019). The BLAST analysis indicated that 504 belongs to *B*. *velezensis* by using the partial *16S rRNA* gene sequences (Li et al., 2019). To further define the phylogenetic status of 504, we sequenced the complete genome of 504, and found that it has a circular chromosome of 3,905,813 base pairs with 46.60% G+C content, 3,853 protein coding sequences (CDSs), 27 rRNA genes, 86 tRNA genes and 217 other non-coding RNA genes (**Figure 1A** and **Supplementary Tables 2**, **3**), as well as a plasmid of 8,564 base pairs with 39.86% G+C content (**Figure 1B**), which is different from *B*. *velezensis* FZB42 and SQR9, two representative model strains of *B. velezensis* (**Supplementary Table 4**). The chromosome and plasmid sequences have been deposited in GenBank under the accession numbers of CP092439 and CP092440, respectively. Further, we executed an average nucleotide identity (ANI) analysis between 504 with eleven sequenced species of *Bacillus* by ANI based on BLAST + (ANIb) and MUMmer (ANIm) applying the online tool JSpeciesWS, respectively. Estimated values of ANIb and ANIm between *B*. *velezensis* 504 and FZB42 were 97.60% [94.03] and 97.77% [94.77], while the ANIb and ANIm values between *B*. *velezensis* 504 and SQR9 were 97.56% [93.97] and 97.75% [89.60], respectively (**Table 1**). The results showed that 504 displayed an ANIb (or ANIm) value over 95% with *B. velezensis* FZB42 and SQR9. All ANI-values (ANIb and ANIm) between 504 and other individual species of *Bacillus* are below the threshold of 95% for species demarcation, indicating that 504 belongs to *Bacillus velezensis*, which is consistent with the result we obtained using the *16S rRNA* gene sequence and alignment.

**Table 1:** ANI analyses between strain 504 and other representative *Bacillus* species.

	Taxon	Starin	1	2	3	4	5	6	7	8	9	10	11	12
**1**	*B. velezensis*	504		97.60 [94.03]	97.56 [93.97]	94.13 [91.92]	93.41 [88.30]	76.34 [74.99]	76.58 [70.18]	76.48 [71.52]	76.82 [69.76]	72.10 [51.46]	76.72 [70.52]	76.51 [69.05]
**2**	*B. velezensis*	FZB42	97.77 [94.77]		98.39 [94.15]	93.95 [88.72]	93.36 [87.34]	76.36 [75.08]	76.57 [75.06]	76.50 [73.73]	76.82 [74.01]	72.11 [54.88]	76.76 [71.89]	76.54 [69.24]
**3**	*B. velezensis*	SQR9	97.75 [89.60]	98.48 [90.24]		93.69 [84.67]	93.08 [83.60]	76.12 [72.29]	76.25 [71.26]	76.14 [71.30]	76.63 [70.88]	71.86 [52.58]	76.63 [68.22]	76.36 [65.80]
**4**	*B*. *siamensis*	KCTC13613	94.43 [92.49]	94.42 [91.81]	94.40 [91.75]		93.27 [88.91]	76.11 [75.93]	76.28 [75.68]	76.28 [74.58]	76.59 [75.17]	71.59 [55.82]	76.42 [72.88]	76.22 [70.60]
**5**	*B*. *amyloliquefaciens*	DSM 7	94.21 [86.81]	94.30 [85.81]	94.23 [86.16]	94.08 [85.30]		76.42 [72.26]	76.65 [72.15]	76.59 [71.52]	77.14 [71.81]	72.11 [54.06]	76.71 [70.45]	76.53 [67.11]
**6**	*B*. *subtilis* subsp. *subtilis*	168	84.44 [24.15]	84.56 [24.48]	84.48 [24.77]	84.37 [24.08]	84.53 [23.87]		86.95 [86.14]	92.23 [85.08]	79.32 [76.18]	72.14 [56.04]	86.83 [83.49]	90.59 [80.24]
**7**	*B*. *halotolerans*	ZB201702	84.33 [25.64]	84.47 [26.07]	84.37 [26.02]	84.06 [25.06]	84.43 [24.97]	87.87 [84.73]		87.78 [85.78]	80.33 [77.49]	72.26 [56.98]	95.83 [88.28]	86.75 [80.70]
**8**	*B*. *subtilis* subsp. *spizizenii*	W23	84.36 [25.54]	84.41 [26.30]	84.19 [26.17]	84.20 [25.03]	84.25 [25.12]	92.89 [89.48]	88.51 [87.80]		79.66 [77.67]	72.15 [58.98]	87.71 [86.24]	92.10 [84.56]
**9**	*B*. *atrophaeus* subsp. *globigii*	BSS	84.14 [24.60]	84.30 [24.49]	84.25 [24.77]	83.70 [24.27]	84.08 [25.16]	84.24 [40.10]	84.61 [46.13]	84.19 [41.89]		72.22 [56.52]	80.09 [75.26]	79.15 [72.12]
**10**	*B*. *licheniformis*	SCDB 14	84.59 [6.17]	84.51 [6.49]	84.54 [6.13]	84.33 [5.92]	85.23 [5.86]	85.07 [6.32]	84.96 [6.99]	85.95 [6.55]	84.37 [6.54]		72.09 [56.93]	72.08 [55.62]
**11**	*B*. *mojavensis*	UCMB5075	84.45 [25.22]	84.54 [25.25]	84.34 [25.83]	84.05 [25.28]	84.48 [24.82]	87.70 [85.29]	96.10 [92.26]	88.44 [85.89]	84.69 [45.34]	84.91 [7.37]		86.64 [83.75]
**12**	*B*. *vallismortis*	Bac111	84.30 [24.51]	84.34 [24.19]	84.15 [24.48]	83.97 [24.92]	84.25 [24.09]	91.23 [87.10]	87.58 [83.99]	92.61 [87.79]	84.17 [40.75]	85.97 [6.68]	87.48 [83.79]	

ANI (Average nucleotide identity) values indicate the pairwise comparisons of given genomic sequences with the genome of strain 504. ANIb and [aligned nucleotides] (%) are shown in the upper right part of the table. ANIm and [aligned nucleotides] (%) are shown in the lower left part of the table. ANIb and ANIm are separated by blue shading. ANIb is calculated with BLAST+, while ANIm is calculated with MUMmer. Values above 95% are highlighted in green.

*B. velezensis* 504 contained complete gene clusters implicated in flagellar assembly (ko02040), bacterial motility proteins (ko02035) and chemotaxis (ko02030), including 56 genes involved in the flagellar structural components (*motA*-*B*, *fliM*, *fliG*, *fliF*), biosynthesis of flagella (*flhA*-*B*, *flhF*-*G*, *fliP*-*R*) and chemotactic signaling protein phospho-CheY (*cheA*-*D*, *CheY*-*P*) (**Supplementary Table 5**), which are required for bacterial survival by regulating the direction of flagellar rotation.

**Figure 2:**
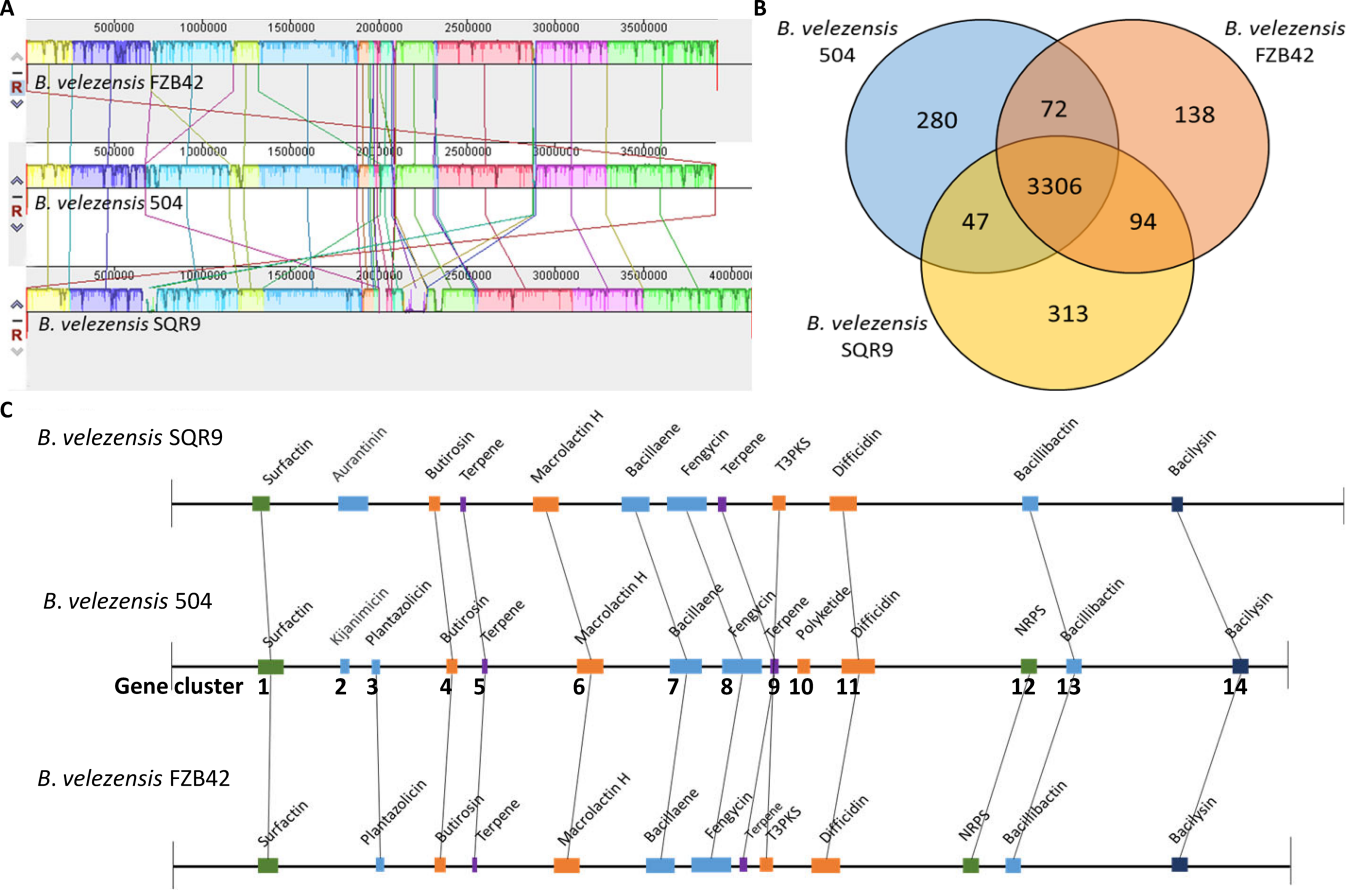
Comparative genomic analysis of *B. velezensis* 504 with other *B. velezensis* strains (*B. velezensis* FZB42 and SQR9). **(A)** Genome-to-genome alignment of *B. velezensis* 504, *B. velezensis* FZB42, and *B. velezensis* SQR9 using a progressive mauve software with *B*. *velezensis* 504 as the reference genome. Similar-colored boxes represent syntenic regions. Boxes below the horizontal line represent regions that are inverted. Rearrangements are depicted with colored lines. **(B)** Venn diagram illustrating the number of orthologous CDS genes that are shared and unique among three strains of *B. velezensis* 504, *B. velezensis* FZB42, and *B. velezensis* SQR9. **(C)** Gene clusters of secondary metabolite biosynthesis in *B. velezensis* 504, *B. velezensis* FZB42, and *B. velezensis* SQR9. The number represented the Jaccard index on the horizontal line. Connected by black lines are the colinear regions. T3PKS, type III PKS; NRPS, non-ribosomal peptide synthetase cluster.

Collinearity analysis of the whole genome between *B. velezensis* 504, FZB42 and SQR9 showed that most genes are highly homologous with direct collinearity, but there were also gene rearrangement phenomena such as flipping and transposition (**Figure 2A**). Moreover, the similarity between *B. velezensis* 504 and FZB42 showed the higher synteny than that of SQR9, which is consistent with the comparison of the general characteristics among their genomes. Core-Pan genome analysis showed that 3306 core genes were conserved in *B. velezensis* 504, FZB42, and SQR9, accounting for 89.23%, 91.58%, and 87.93% of the genes in the genomes, respectively (**Figure 2B**). Total 280 genes unique to *B. velezensis* 504 don't exist in FZB42 and SQR9. 71.07% of these genes code for hypothetical proteins, 14.29% have predictive functions, 8.57% have non-predictive functions, and 5.36% code for phage portal proteins (**Supplementary Table 6**). 44 unique genes were predicted to encode clusters of secondary metabolites (**Supplementary Table 7**), of which 10 genes were predicted to encode kijanimicin (3.57%), 6 genes were predicted to encode fengycin (2.14%), and 10 genes were unknown function descriptions. (3.57%).

Secondary metabolite clusters of *B. velezensis* 504 were compared with *B. velezensis* FZB42 and SQR9 using antibiotics and secondary metabolite analysis shell (anti-SMASH). The result revealed that the genome of *B*. *velezensis* 504 contains fourteen candidate gene clusters involved in nonribosomal synthesis of lipopeptides (surfactin, fengycin and bacillibactin), polyketides (difficidin, bacillaene, macrolactin H, and kijanimicin), dipeptide antibiotics (bacilysin), ribosomal synthesis of bacteriocins (plantazolicin), saccharide synthesis of butirosin, and other four unknown terpenes, polyketide and non-ribosomal peptide synthetases (**Figure 2C** and **Supplementary Table 8**). *B. velezensis* 504 contains several clusters that share different structures with FZB42 and SQR9, and lacks *yx01*, *aat*, *ycxC*, and *ycxD* in the Cluster 1 encoding surfactin (**Supplementary Table 9**). Cluster 2 involved in kijanimicin synthesis and Cluster 10 involved in polyketide synthesis exist only in *B. velezensis* 504, Cluster 3 and Cluster 12 encoding plantazolicin and NRPS exist in both *B. velezensis* 504 and FZB42, and other ten (Clusters 1, 4-9, 11, 13 and 14) conservatively present in all three strains (**Figure 2C** and **Supplementary Table 9**). Six clusters involved in macrolactin H, bacillaene, fengycin, bacillibactin, difficidin and bacilysin synthesis, share 100% homology with that of *B. velezensis* FZB42 (**Supplementary Table 8**). These bioactive molecules produced by *B. velezensis* FZB42 have been reported to be effective against phytopathogenic fungi and bacteria, indicating that *B*. *velezensis* 504 has the potential to control fungal and bacterial diseases (Rabbee et al., 2019). Difficidin and bacilysin have been reported to be the essential antibacterial agents produced by *B. velezensis* FZB42 to control BB and BLS of rice, two major rice diseases caused by *Xoo* and *Xoc*, respectively (Wu et al., 2015). Therefore, we speculated that difficidin and bacilysin are major extracellular active molecules of *B*. *velezensis* 504 to antagonize against *Xoc* RS105.

### 3.2 Comparative transcriptional profile analysis to define the response of *Xoc* to the cell-free supernatants of *B. velezensis* 504

**Figure 3:**
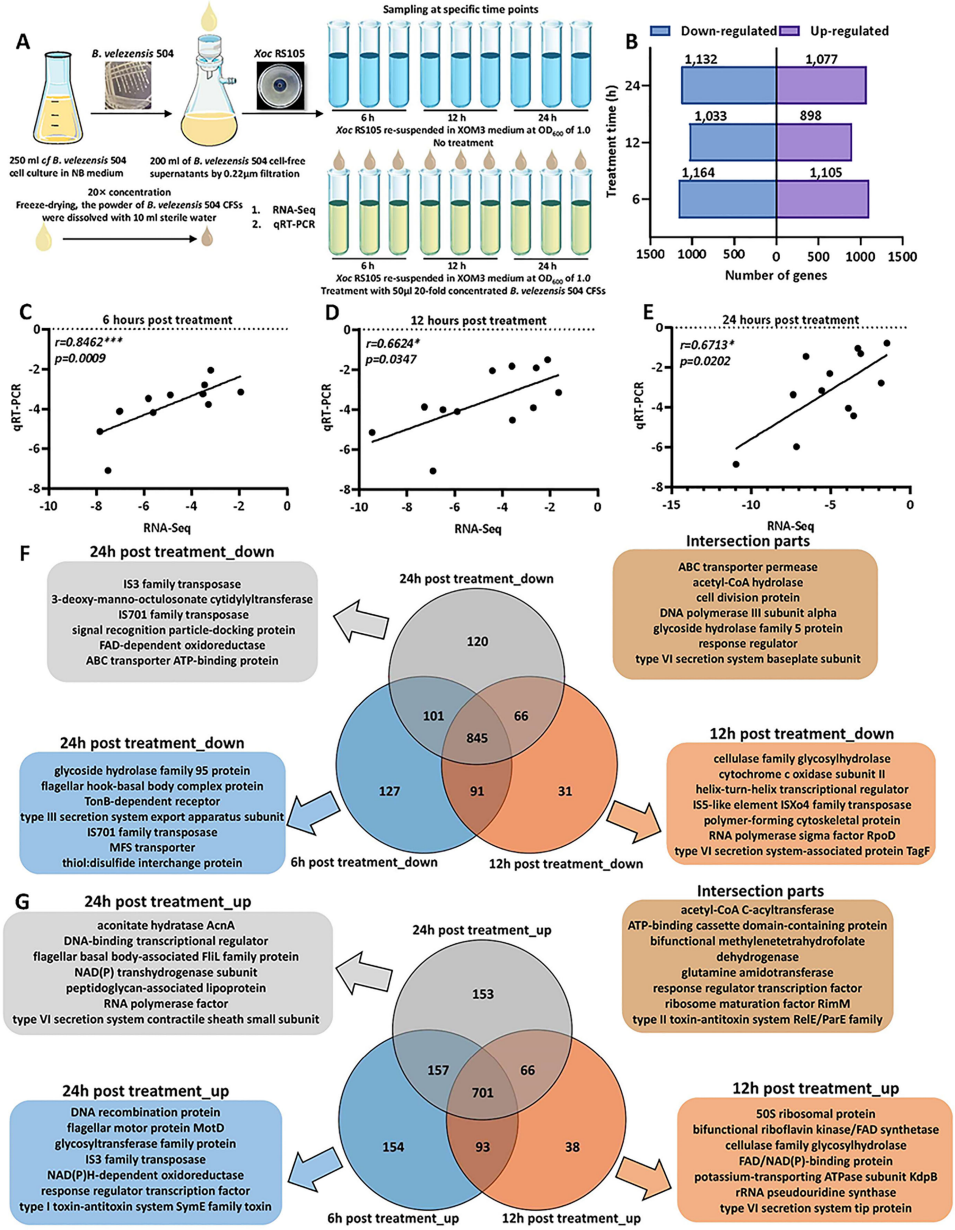
Overview of transcriptome of RS105 under *B. velezensis* 504 CFSs treatment. **(A)** Schematic presentation of the transcriptome assay of *B. velezensis* 504 CFS-treated RS105. **(B)** Bar chart of downregulated and upregulated DEGs distribution between RS105 and 504-treated RS105 at three distinct time points. **(C–E)** Gene expression patterns obtained through RNA-Seq and qRT-PCR validation have been compared. The Y-axis displays the Log_2_FCs of the DEGs obtained using qRT-PCR validation, whereas the X-axis represents the Log_2_FCs of the DEGs obtained using RNA-Seq. The equation described the correlation between X and Y. **(F)** Venn diagram showing the overlapping down-regulated DEGs at three distinct time points. **(G)** Venn diagram showing the overlapping up-regulated DEGs at three distinct time points.

Our study confirmed that 50 µL 20-fold concentrated cell-free supernatants (CFSs) of *B. velezensis* 504 exhibited prominent antibacterial activities against *Xoc* RS105 (**Figure 3A** and **Supplementary Figure 4B**), suggesting that *B. velezensis* 504 prevented the visible growth of *Xoc*, most likely by synthetic metabolites that were diffusible across the 0.22 μm filter membrane. To explore the molecular mechanism underlying antibacterial effect of *B*. *velezensis* 504 on *Xoc* RS105, we first determined the global transcriptional response of *Xoc* RS105 to the CFSs of *B*. *velezensis* 504 compared to that induced by XOM3, an artificial *hrp* inducing medium. We isolated total RNA from *Xoc* RS105, at 6 hr, 12 hr, and 24 hr post induction (hpi) by the CFSs of *B*. *velezensis* 504 (20 times the concentration) dissolved in XOM3 (hereafter referred to as 504) and by XOM3 itself (hereafter called CK), respectively (**Figure 3A**). High-quality raw reads (Q20>98%) were mapped to the reference genome of *Xoc* RS105 (RefSeq assembly accession: GCF_001042875.1) in the NCBI database (**Supplementary Tables 10**, **11**).

Differentially expressed genes (DEGs) were defined using a P-value adjusted for the false-discovery rate (P_adj_ **<**0.01), and a moderated log2-transformed fold change in gene expression (mod_lfc)>1 to define upregulated genes, and mod_lfc<-1 to define downregulated genes. We observed 2,269, 1,931, 2,209 *Xoc* RS105 DEGs in response to the CFSs of *B*. *velezensis* 504 at 6 hpi, 12 hpi and 24 hpi, respectively (**Figure 3B**). To verify the reliability of the RNA-Seq data, we selected twelve interested genes (*hpa1*, *hrpE*, *hrcC*, *rpsQ*, *nuoF*, *nuoL*, *glnA*, *lpdA*, *katB*, *fliM*, *flgL*, and *cheY*) related to ribosome (ko03010), oxidative phosphorylation (ko00190), glycolysis/gluconeogenesis (ko00010), flagellar assembly (ko02040), bacterial chemotaxis (ko02030), two-component system (ko02020) and T3SS to evaluate the abundance for validation (**Supplementary Table 12**). Gene expression levels that carried out by qRT-PCR showed agreement with the changes detected by RNA-Seq at 6, 12, and 24 h-post treatment (spearman correlation coefficient of 0.8462, 0.6624, and 0.6713, *p* value<0.05), indicating the reliability of the transcriptome results (**Figures 3C–E** and **Supplementary Table 13**). Of these genes, 1,164, 1,033 and 1,132 genes were down-regulated, while 1,105, 898 and 1,077 genes were up-regulated (**Figure 3B** and **Supplementary Tables 14**, **15**). A total of 1,546 genes were found to be differentially expressed at all three time points, of which 845 were down-regulated and 701 were up-regulated (**Figures 3F, G**). The significantly down-regulated genes identified function associated with ABC transporter permease, cell division proteins, DNA poltmerase III subunit alpha, and type VI secretion system baseplate subunit (**Figure 3F**). The significantly up-regulated genes identified function associated with acetyl-CoA C-acyltransferase, ATP-binding cassette domain-containing proteins, glutamine amidotransferase and response regulator transcription factors (**Figure 3G**). A comparison of these gene sets identified 291 DEGs (154 up-regulated, 127 down-regulated), 69 DEGs (38 up-regulated, 31 down-regulated), and 273 DEGs (153 up-regulated, 120 down-regulated) with a 6-hr, 12-hr, and 24-hr dependent expression pattern, respectively (**Figures 3F, G** **)**. We concluded that 2743 genes (1381 down-regulated, 1362 up-regulated) corresponding to 77% of *Xoc* RS105 coding sequences (CDSs) were differentially expressed by the CFSs of *B*. *velezensis* 504.

Further, we performed comparative RNA-seq of *Xoc* RS105 induced by the CFSs of *B*. *velezensis* 504 at 6 hr, 12 hr and 24 hr. This led to the identification of 435 DEGs down-regulated and 316 DEGs up-regulated with significant changes in expression during treatment by the CFSs of *B*. *velezensis* 504 (**Supplementary Figure 1**). A comparison of these gene sets found that 9 DEGs were consistently down-regulated over three time periods while 6 DEGs were consistently up-regulated (**Supplementary Table 16**).

### 3.3 Metabolism, transmembrane transport, cell motility, DNA translation and signal transduction are affected by the CFSs of *B*. *velezensis* 504

To further investigate functions of genes regulated by the CFSs of *B*. *velezensis* 504, we performed the Gene Ontology (GO) enrichment analysis. The DEGs were enriched in the cellular process (GO:0009987), bacterial-type flagellum basal body (GO:0009425) and flagellum-dependent cell motility (GO:0001539), chemotaxis (GO:0006935), organic substance transport (GO:0071702), carbohydrate transmembrane transporter activity (GO:0015144), transcription regulator activity (GO:0140110), sigma factor activity (GO:0016987), hydrolase activity (GO:0016787) acting on carbon-nitrogen, pyrophosphate activity, nucleoside triphosphatase activity, and dTTP metabolic process (**Supplementary Figure S2**). In the KEGG analysis, the *B. velezensis* 504 CFSs-induced DEGs were significantly enriched in 88 pathways including flagellar assembly (ko02040), two-component system (ko02020) and bacterial chemotaxis (ko02030) ranking the top three rich factors, followed by ribosome (ko03010), lipopolysaccharide biosynthesis (ko00540), tyrosine metabolism (ko00350), lysine biosynthesis (ko00300), as well as glyoxylate and dicarboxylate metabolism (ko00630) (**Supplementary Figure S3** and **Supplementary Table 17**).

**Figure 4:**
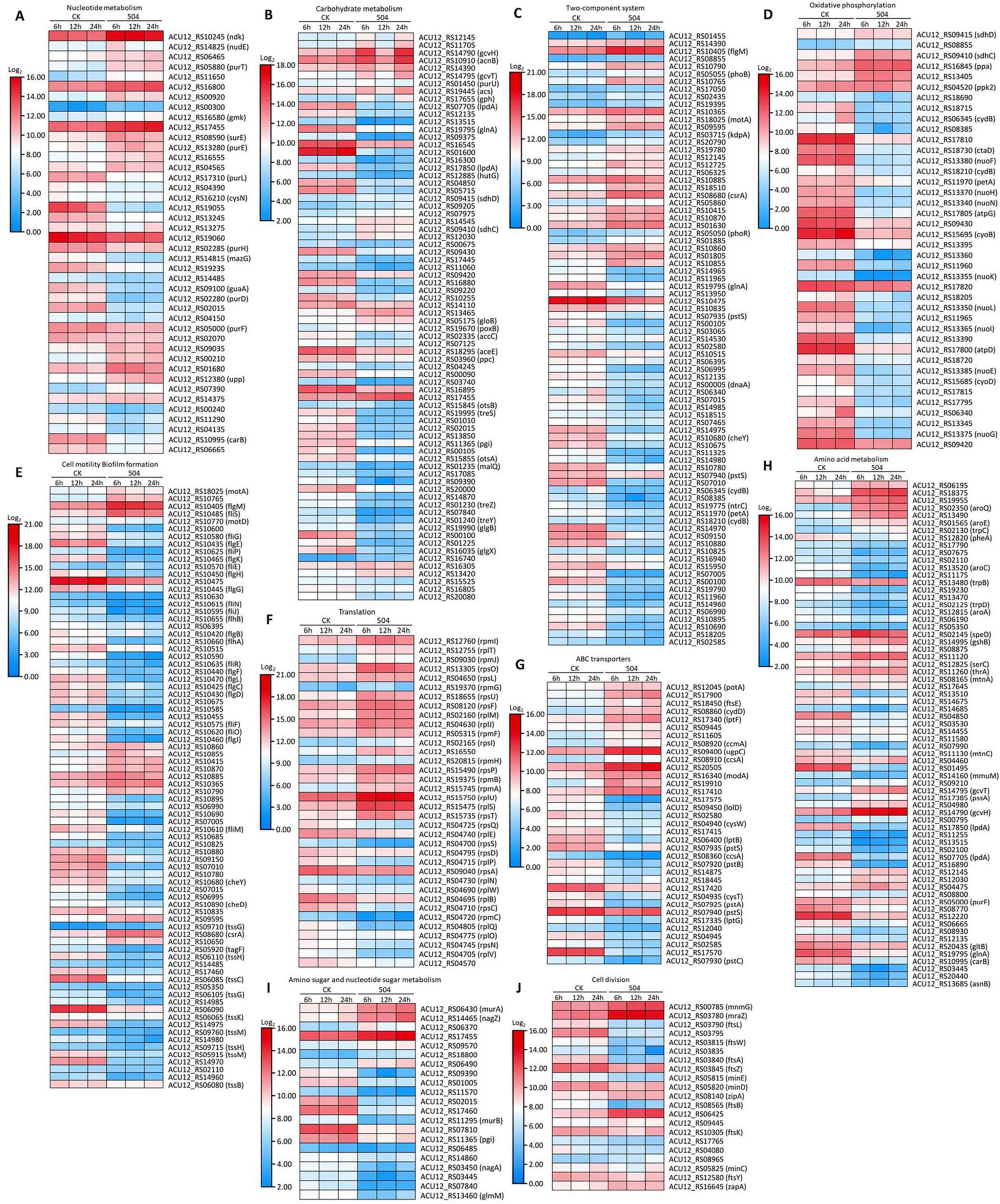
Defining the transcriptional response of RS105 to the CFSs of *B. velezensis* 504. **(A)** Heatmap showing the temporal pattern of the relative transcript abundance of nucleotide metabolism genes between RS105 and the CFSs of 504-treated RS105. **(B)** Heatmap showing the temporal pattern of the relative transcript abundance of carbohydrate metabolism genes between RS105 and the CFSs of 504-treated RS105. **(C)** Heatmap showing the temporal pattern of the relative transcript abundance of two-component genes between RS105 and the CFSs of 504-treated RS105. **(D)** Heatmap showing the temporal pattern of the relative transcript abundance of oxidative phosphorylation genes between RS105 and the CFSs of 504-treated RS105. **(E)** Heatmap showing the temporal pattern of the relative transcript abundance of cell motility and biofilm formation genes between RS105 and the CFSs of 504-treated RS105. **(F)** Heatmap showing the temporal pattern of the relative transcript abundance of translation genes between RS105 and the CFSs of 504-treated RS105. **(G)** Heatmap showing the temporal pattern of the relative transcript abundance of ABC transporters genes between RS105 and the CFSs of 504-treated RS105. **(H)** Heatmap showing the temporal pattern of the relative transcript abundance of Amino acid metabolism genes between RS105 and the CFSs of 504-treated RS105. **(I)** Heatmap showing the temporal pattern of the relative transcript abundance of amino sugar and nucleotide sugar metabolism genes between RS105 and the CFSs of 504-treated RS105. **(J)** Heatmap showing the temporal pattern of the relative transcript abundance of cell division genes between RS105 and the CFSs of 504-treated RS105.

**Figure 5:**
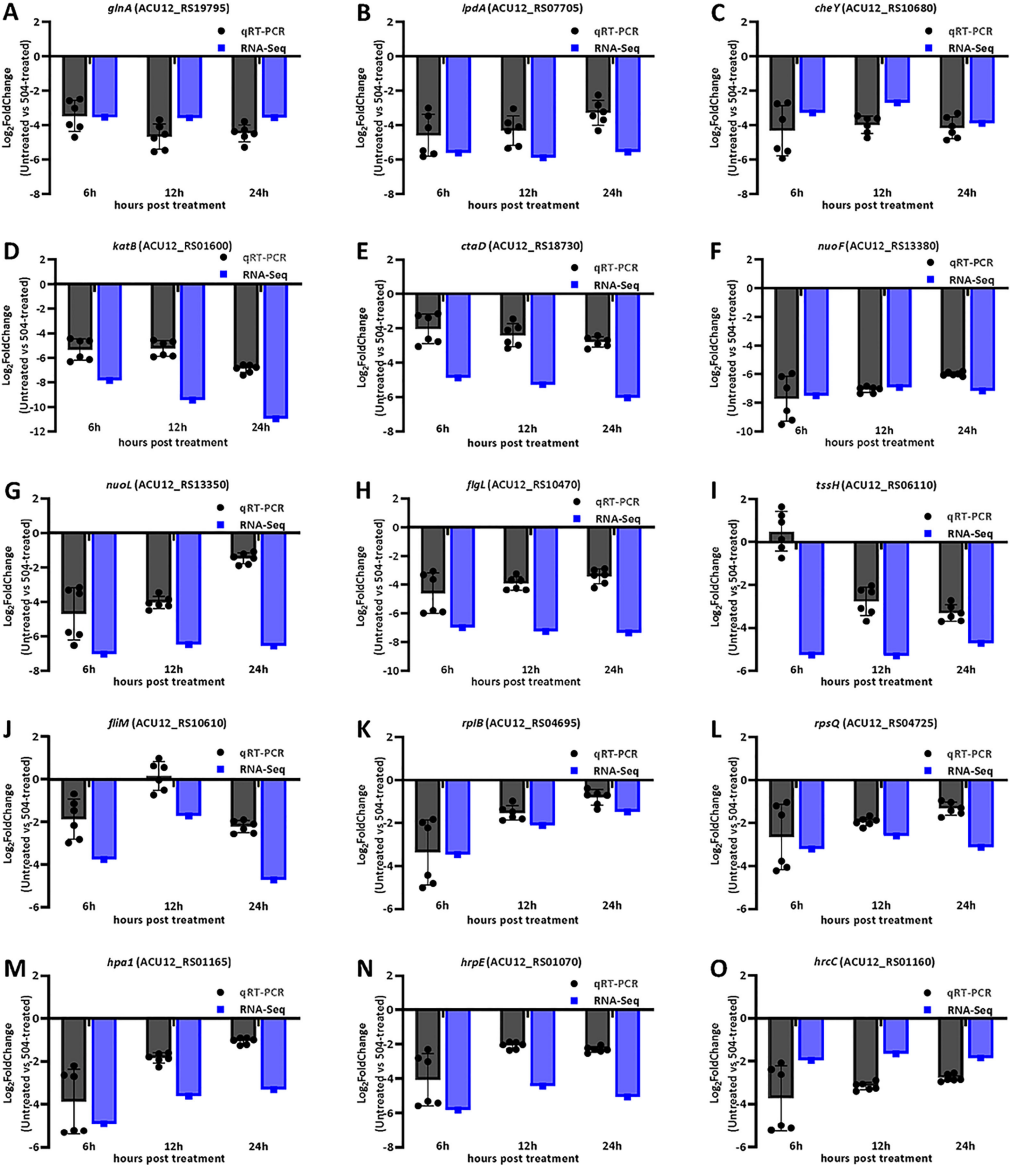
qRT-PCR assays for gene expression in RS105 treated with *B. velezensis* 504 CFSs. **(A–O)** The X-axis represents the 6, 12, and 24hpt. The Y-axis represents the Log_2_FoldChange (Untreated vs the CFSs of 504-treated) of relative expression level determined by RNA-Seq and qRT-PCR using *rpoD* and *gyrB* as an endogenous control. All experiments were repeated three times with consistent results.

To explore possible antagonistic mechanism of the *B. velezensis* 504 CFSs against *Xoc* RS105, we observed the expression of some genes associated with metabolism, cellular process, and signal transduction processing from DEGs (**Supplementary Table 18**). Transcript levels of 20 out of 42 genes involved in nucleotide metabolism, such as *ACU12_RS19055* predicted to encode ibonucleotide-diphosphate reductase subunit beta, *ACU12_RS13275* predicted to encode 5-(carboxyamino) imidazole ribonucleotide synthase, *ACU12_RS02015* predicted to encode phosphomannomutase, and *guaA* predicted to encode glutamine-hydrolyzing GMP synthase, were down-regulated by treatment with the CFSs of *B. velezensis* 504 (**Figure 4A**). Furthermore, *B. velezensis* 504 CFSs affected the expression of genes involved in energy production and transformation pathway in RS105, including glyoxylate and dicarboxylate metabolism (ko00630), butanoate metabolism (ko00650), pyruvate metabolism (ko00620) and other biochemical reaction processes (**Figure 4B**). RNA-Seq analysis found that *glnA* (*ACU12_RS19795*), *lpdA* (*ACU12_RS17850*) and *katB* (*ACU12_RS01600*) were down-regulated at 6, 12, 24 hpi under the treatment of *B. velezensis* 504 CFSs, which is in agreement with the qRT-PCR analysis (**Figures 5A, B, D**). Transcript levels of 14 out of 21 genes involved in amino sugar and nucleotide sugar metabolism, as well as 43 out of 68 genes involved in amino acid metabolism such as phenylalanine, tyrosine and tryptophan biosynthesis, were down-regulated by the CFSs of *B. velezensis* 504 at three time points (**Figures 4H, I**). These results indicate that the genes related to the metabolisms of nucleotide, carbohydrate, amino sugar and nucleotide sugar, and amino acid are repressed by the treatment of *B. velezensis* 504 CFSs.

Transcript levels of 35 out of 40 genes involved in oxidative phosphorylation were significantly down-regulated by the CFSs of *B. velezensis* 504 at 6 hpi, 12 hpi, and 24 hpi (**Figure 4D**). The expression pattern of *ctaD* (*ACU12_RS18730*), *nuoF* (*ACU12_RS13380*), and *nuoL* (*ACU12_RS13350*) by qRT-PCR analysis was coincident with the RNA-Seq data (**Figures 5E–G**). After *B. velezensis* 504 CFSs treatment, DEGs were mainly enriched in flagellar assembly (ko02040) and bacterial chemotaxis (ko02030), and their expression was significantly down-regulated, including flagellar and flagellin related genes (i.e., *flgB*-*flgH*, *flgJ*-*flgL*, *flhA*-*flhB*, *fliE*-*fliG*) (**Figure 4E**). The expression of *flgL, fliM, and cheY* in *Xoc* RS105 were downregulated by the *B. velezensis* 504 CFSs at 6 hpi, 12 hpi, and 24 hpi using qRT-PCR analysis. (**Figures 5C, H, J**), suggesting that *B. velezensis* 504 can inhibit flagellar assembly processing of *Xoc* RS105.

Fifteen genes (*rpsQ*, *rplE*, *rpsS*, *rpsD*, *rplP*, *rpsA*, *rplN*, *rplW*, *rplB*, *rpsC*, *rpmC rplQ*, *rplO*, *rpsN*, and *rplV*) in the ribosomal operon were down-regulated in RS105 treated with the CFSs of *B. velezensis* 504 (**Figure 4F**). *rplB* and *rpsQ* were decreased in 3.47- and 3.21-fold, 2.11- and 2.59-fold, 1.48- and 3.13-fold in RS105 at 6 hpi, 12 hpi, and 24 hpi with the addition of *B. velezensis* 504 CFSs, respectively (**Figures 5K, L**) suggesting that *B. velezensis* 504 can inhibit growth of *Xoc* RS105 by reducing the genetic information processing. Treatment of *B. velezensis* 504 CFSs caused significantly differential expression of 21 genes involved in cell division in *Xoc* RS105. (**Figure 4J**). Fourteen cell division (*minCDE* system genes, *ftsA*, *ftsB*, *ftsL*, *ftsW*, *ftsY*, etc.) were downregulated in RS105 treated with *B. velezensis* 504 CFSs at 6 h (**Figure 4J**). We also observed that expression levels of 21 out of 35 genes involved in ABC transporters, as well as 50 out of 81 genes involved in two-component systems were significantly down-regulated by the CFSs of *B. velezensis* 504 at 6 hpi, 12 hpi, and 24 hpi (**Figures 4C, G**). Taken together, the results indicating that *B*. *velezensis* 504 may exert antibacterial activity by affecting the entire metabolic pathway, transmembrane transport, cell motility, DNA translation and signal transduction.

### 3.4 Expression of some major virulence related genes is significantly affected in response to the CFSs of *B. velezensis* 504

**Figure 6:**
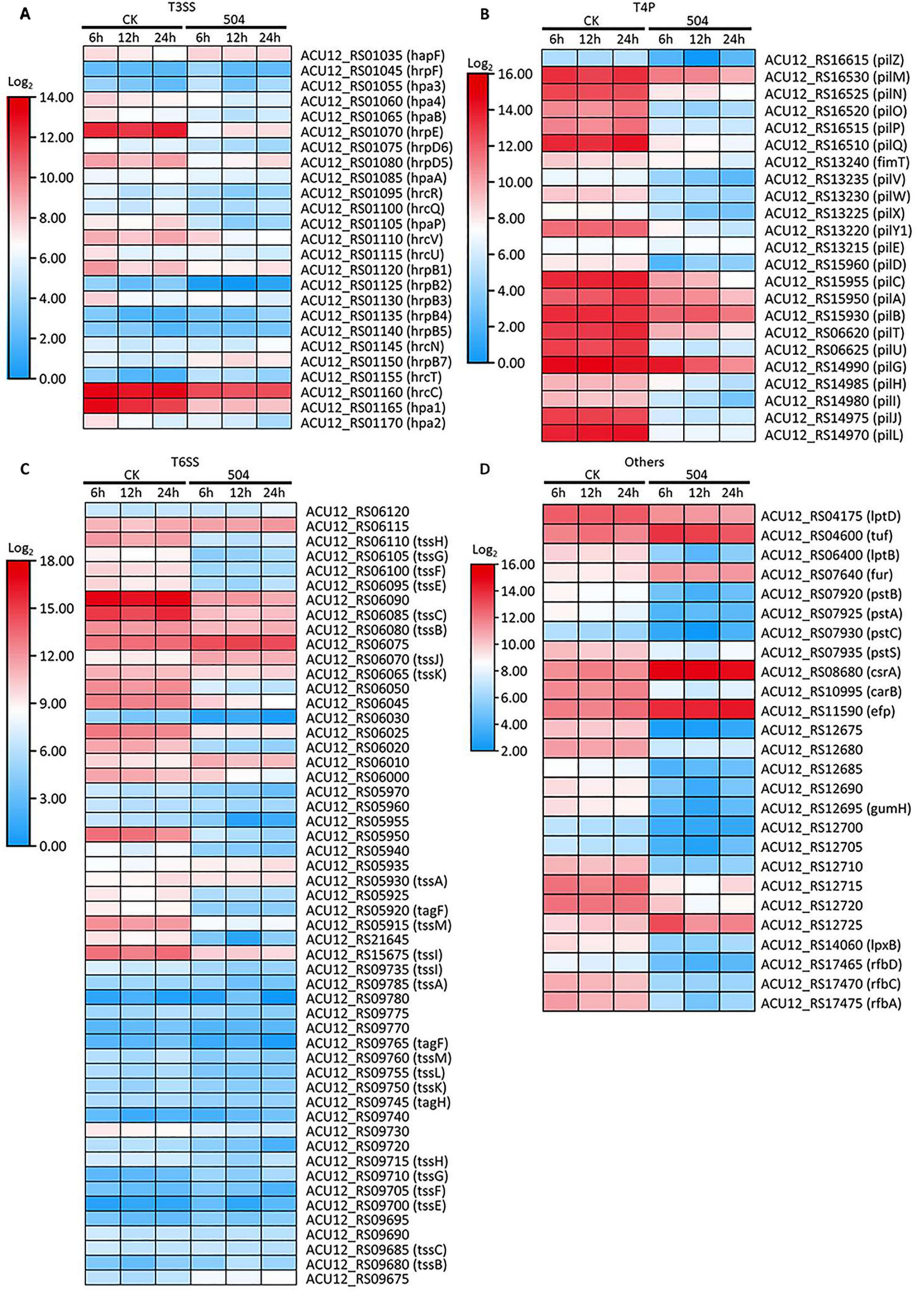
Defining the transcriptional response of RS105 virulence genes to the CFSs of *B. velezensis* 504 treatment. **(A)** Heatmap showing the temporal pattern of the relative transcript abundance of T3SS genes between RS105 and the CFSs of 504-treated RS105. **(B)** Heatmap showing the temporal pattern of the relative transcript abundance of T4P genes between RS105 and the CFSs of 504-treated RS105. **(C)** Heatmap showing the temporal pattern of the relative transcript abundance of T6SS genes between RS105 and the CFSs of 504-treated RS105. **(D)** Heatmap showing the temporal pattern of the relative transcript abundance of other genes related with virulence between RS105 and the CFSs of 504-treated RS105.

The successful pathogenesis by *Xoc* in host rice tissues is dependent on some critical virulence factors including type III secretion system (T3SS), lipopolysaccharides (LPS), exopolysaccharides (EPS), adhesins, extracellular enzymes, toxins, cell motility, and so on (Buttner and Bonas, 2010). To further explore the biocontrol mechanisms of *B. velezensis* 504, we observed the expression of some major virulence related genes from DEGs (**Supplementary Table 19**). We observed the expression of 25 T3SS genes (*hrc, hrp* and *hpa*) encoding the T3SS secretion system of *Xoc* RS105, and found that 14 genes (*hpa2, hpa1, hrcC, hrpB3, hrpB2, hrpB1, hrcU, hpaP, hrcR, hrpD5, hrpD6, hrpE, hpaB* and *hpa4*) were down-regulated at 6 hpi by the CFSs of *B. velezensis* 504, 7 genes (*hpa1, hrcC, hrcV, hpaP, hrpD5, hrpE, and hpa4*) were down-regulated at 12 hpi, and 8 genes (*hpa1, hrcC, hrcV, hpaP, hrpD5, hrpD6, hrpE*, and *hpa4*) were down-regulated at 12 hpi, whereas 6 genes (*hpa1, hrcC, hpaP, hrpD5, hrpE*, and *hpa4*) were down-regulated at all three time periods (**Figure 6A**). This result was consistent with that by the qRT-PCR analysis of *hpa1*, *hrpE* and *hrcC* (**Figures 5M–O**). In addition, we found that genes *gspD* (*ACU12_RS17240*), *gspN* (*ACU12_RS17250*), *gspG* (*ACU12_RS17285*) encoding the critical components of type II secretion system (T2SS) of *Xoc* RS105 that is responsible for the secretion of extracellular enzymes were downregulated at 6 hpi, 12 hpi and 24 hpi by the CFSs of *B. velezensis* 504. These results indicate that the CFSs of *B. velezensis* 504 can weaken the functions of T3SS and T2SS of *Xoc* RS105.

Bacterial attachment on host tissues relies on some adhesins that are classified into fimbrial and nonfimbrial adhesins (Li et al., 2020b). *Xoc* can use a polar flagellum and dynamic type IV pili (T4P) to contact surfaces of the host rice cells and can move across the surface using T4P-mediated twitching motility (Wei et al., 2021). We found that 23 T4P-related genes, except *pilE*, were down-regulated at 6 hpi, 12 hpi and 24 hpi by the CFSs of *B. velezensis* 504, among which the genes *pilMNOP* encoding the alignment complex, the genes *pilVWX* encoding the minor pilins, the gene *pilY1* encoding the envelope protein PilY1 (adhesin), the gene *pilD* encoding a pre-pilin peptidase PilD, the gene *pilU* encoding a putative ATPase *PilU* were most significantly down-regulated (**Figure 6B**). This indicates that the CFSs of *B. velezensis 504* can attenuate the function of T4P of *Xoc* RS105.

Type VI secretion system (T6SS) in *Xanthomonas* can directly inject toxins and effectors into cells of eukaryotic and prokaryotic targets by a contact apparatus, and participates in bacterial virulence and competition (Montenegro Benavides et al., 2021). We observed 53 genes associated with T6SS, among which 29 genes were significantly down-regulated, and some genes had two copies, such as *tssH*, *tssG*, *tssF*, *tssE*, *tssC*, *tssB*, *tssA*, *tagF*, tssM and *tssI*, etc. The expression levels of one copy of these genes such as *tssH* (*ACU12_RS06110*), *tssG* (*ACU12_RS06105*), *tssF* (*ACU12_RS06100*), *tssE* (*ACU12_RS06095*), *tssM* (*ACU12_RS05915*) were significantly reduced in RS105 treated with the CFSs of *B. velezensis* 504 (**Figure 6C**). *tssM* (*ACU12_RS05915* and *ACU12_RS09760*) and *tssH* (*ACU12_RS06110*) associated with biofilm formation (ko02025), were down-regulated in 3.71-, 1.58- and 5.27-fold, 4.08-, 0.36- and 5.30-fold, and 3.73-, 2.33-, and 4.71-fold in Xoc RS105 at 6 hpi, 12 hpi, and 24 hpi by treatment of the CFSs of *B. velezensis* 504, respectively (**Figure 6C**). We chose the *tssH* gene for qRT–PCR analysis, the expression of it was down-regulated 2.77-fold, and 3.32-fold in *Xoc* RS105 at 12 hpi, and 24 hpi by the CFSs of *B. velezensis* 504, respectively (**Figure 5I**).

We found that the genes (*lptD*, *lptB*, and *lpxB*) predicted to encode LPS-assembly protein, LPS export ABC transporter, and lipid-A-disaccharide synthase, respectively, with the addition of the genes of *gum* cluster (from *ACU12_RS12675* to *ACU12_RS12725*) associated with the EPS or LPS biosynthetic pathways were significantly down-regulated at 6 hpi, 12 hpi and 24 hpi by the CFSs of *B. velezensis* 504 (**Figure 6D**). The three genes (*rfbA*, *rfbC* and *rfbD*) associated with the EPS synthesis were also down-regulated at 6 hpi, 12 hpi and 24 hpi. The four genes (*pstA*, *pstB*, *pstC* and *pstS*) predicted to encode phosphate ABC transporter were depressed at 6 hpi, 12 hpi and 24 hpi by the CFSs of *B. velezensis* 504 (**Figure 6D**). Downregulation of the *carB* gene, a known virulence gene in *Xanthomonas* spp., predicted to encode carbamoyl-phosphate synthase large subunit was also observed at three time points. However, two known virulence genes (*fur* and *csrA*) predicted to encode ferric iron uptake transcriptional regulator and carbon storage regulator CsrA (or RsmA), respectively, were induced at 6 hpi, 12 hpi and 24 hpi by the CFSs of *B. velezensis* 504 (**Figure 6D**). In addition, the genes *efp* and *tuf* predicted to encode elongation factor P and Tu, respectively, were also induced at three time points (**Figure 6D**). Taken together, these results suggest that *B*. *velezensis* 504 may exert biocontrol effects by down-regulating key virulence genes associated with T3SS, T2SS, T4P, T6SS, EPS and LPS productions, and other virulence factors.

### 3.5 *B. velezensis* 504 is a potential biocontrol agent against bacterial blight of rice

**Figure 7:**
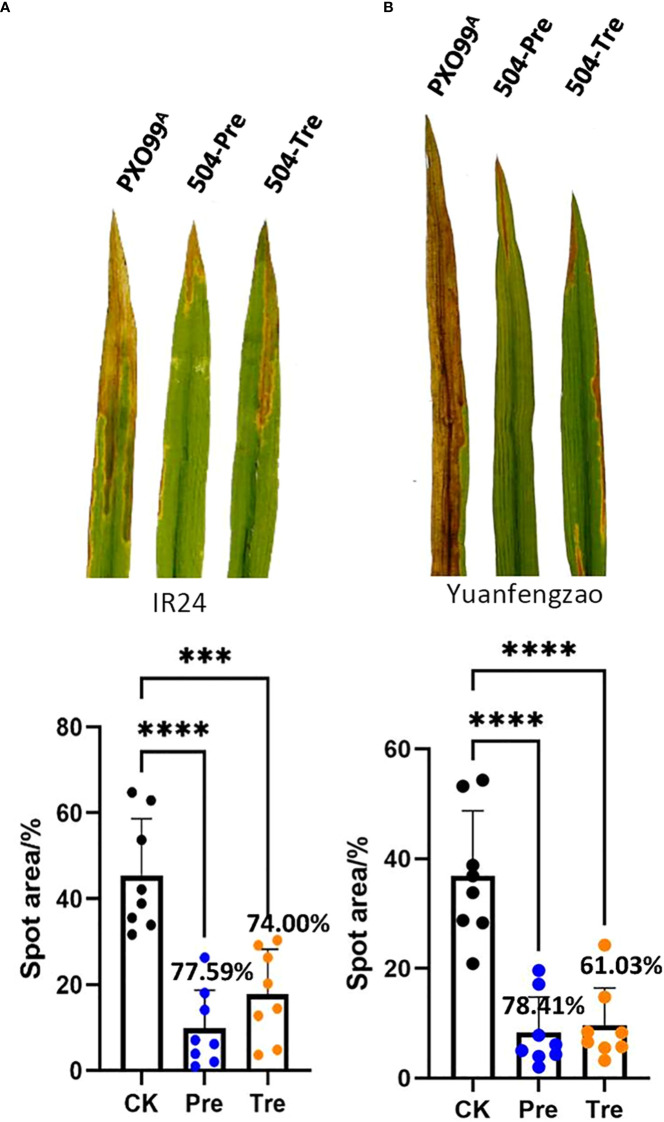
Efficacy of *B. velezensis* 504 for control of *Xoo* PXO99^A^ in the field. The field trials were performed using the susceptible cultivars including indica rice IR24 **(A)** and japonica cultivar Yuanfengzao **(B)**. The prevention (Pre) and treatment (Tre) strategies were executed as follows: *Xoo* PXO99^A^ only (Control), rice leaves sprayed with *B. velezensis* 504 12 h before inoculation with *Xoo* PXO99^A^ suspension (504-Pre), and 12 h after inoculation with *Xoo* PXO99^A^ suspension (504-Tre). The bacterial blight disease of rice (BB) severity was investigated after 15 days. Data points represent means ± SD (n=8 independent leaves). The significant differences at ***P< 0.001 and at ****P< 0.0001.

Our previous results showed that *B*. *velezensis* 504 displayed significant antagonistic activity against *Xoo* and *Xoc*, and demonstrated that *B*. *velezensis* 504 could effectively prevent BLS (Li et al., 2019). We found that the CFSs of *B*. *velezensis* 504 can effectively inhibit the growth of *Xoo* PXO99^A^ (**Supplementary Figure 4A**). To further investigate the biocontrol potential of *B*. *velezensis* 504 against BB of rice caused by *Xoo*, we performed the field trials using three susceptible cultivars including indica rice IR24, japonica cultivar Nipponbare, and Yuanfengzao, a highly susceptible indica rice variety in Hunan province of China. The prevention (Pre) and treatment (Tre) strategies were executed as follows: *Xoo* PXO99^A^ only were used as control (CK), rice leaves sprayed with *B*. *velezensis* 504 12 h before inoculation with *Xoo* PXO99^A^ suspension (504-Pre), and 12 h after inoculation with *Xoo* PXO99^A^ suspension (504-Tre). The BB disease severity by all treatments were investigated 15 days post inoculation. We found that *B*. *velezensis* 504 exhibited an effective biocontrol effect on IR24. Compared with the control, the 504-Pre and 504-Tre treatments dramatically reduced the severity of BB on IR24 with relative control efficiencies of 77.59% and 74.00%, respectively, in paddy fields (**Figure 7A**). The relative control efficiencies by the 504-Pre and 504-Tre treatments were 76.75% and 76.54% on Nipponbare (**Supplementary Figures 4C, D**), respectively, followed by Yuanfengzao with relative control efficiencies 78.41% and 61.03% (**Figure 7B**), indicating that there is no significant difference between the 504-Pre and 504-Tre treatments on Nipponbare and Yuanfengzao. Taken together, these results indicate that *B*. velezensis 504 is a promising biocontrol agent for BB of rice.

### 3.6 *B. velezensis* 504 can efficiently antagonize against some important plant pathogenic fungi and promote plant growth

**Figure 8:**
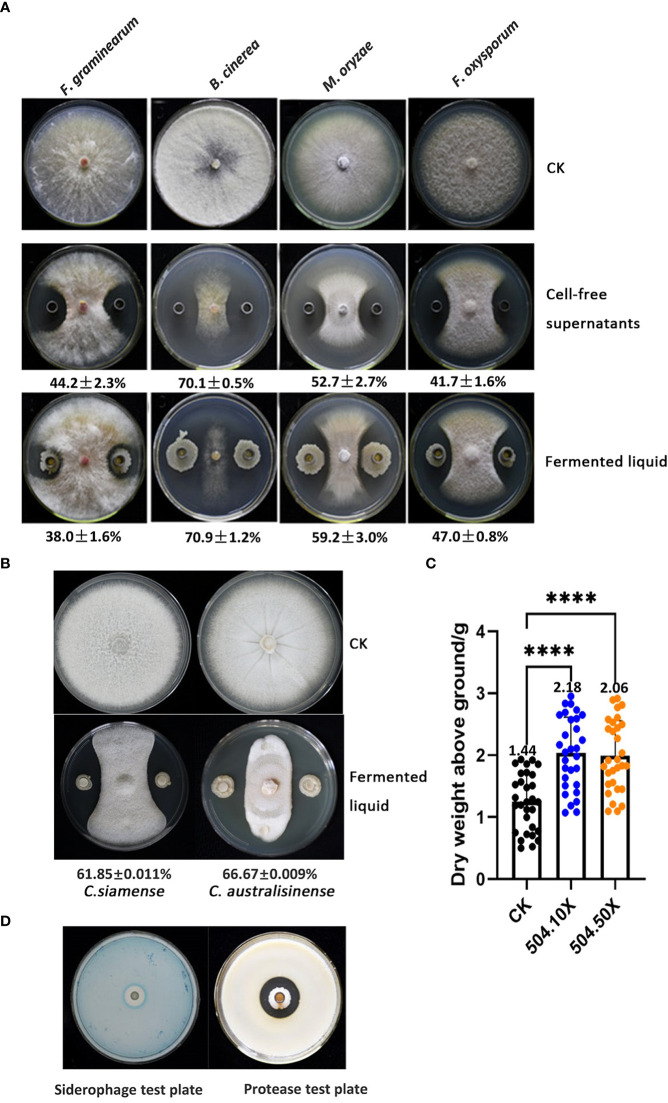
*B. velezensis* 504 can efficiently antagonize against some important plant pathogenic fungi and promote plant growth. **(A)** Determination of *B. velezensis* 504 antagonistic activity against *F. graminearum*, *B. cinerea*, *M*.*oryzae*, *F. oxysporum* in co-cultured assays. Sterile supernatants and fermented liquid of *B. velezensis* 504 were used as treatment, blank was used as control (CK). Three independent biological experiments were performed with similar results. **(B)** Determination of *B. velezensis* 504 antagonistic activities against *C. siamense* and *C. australisinense* in co-cultured assays. **(C)** Effect of *B. velezensis* 504 on growth-promotion of pak choi. **(D)** Siderophores and protease production of *B. velezensis* 504. The significant differences at ****P< 0.0001.

Numerous *B*. *velezensis* strains have been reported to exhibit antagonistic activity against some important plant pathogenic fungi of the genus *Rhizoctonia* and *Fusarium*. We tested the inhibitory effect of *B*. *velezensis* 504 on four fungal pathogens including *Botrytis cinerea*, *Magnaporthe oryzae*, *F*. *graminearum* and *F. oxysporum* on agar plates using Oxford cups whose bottoms were filled up by bacterial suspensions or the CFSs of *B*. *velezensis* 504. The results showed that all the fungal strains spread over the entire surface of the petri dishes in the controls, whereas the growth of their hyphae was obviously suppressed in the presence of either bacterial suspensions or the CFSs of *B*. *velezensis* 504 (**Figure 8A**). *B*. *cinerea*, the etiological agent of gray mold, is the pathogenic fungus most influenced by *B*. *velezensis* 504 with an inhibition rate of 70.1% and 70.9% corresponding to the CFSs and bacterial suspensions treatments, respectively, followed by *M*. *oryzae* causing rice blast disease, with an inhibition rate of 52.7% and 59.2% relative to the CFSs and bacterial suspensions treatments (**Figure 8A**). *F*. *graminearum* and *F. oxysporum* were less sensitive to *B*. *velezensis* 504 and formed smaller inhibition zones, although they also were suppressed with an approximate inhibition rate of 40% (**Figure 8A**). In addition, we evaluated the antifungal activity of *B. velezensis* 504 against *Colletotrichum siamense* and *C*. *australisinense* that are thought to be the two dominant pathogenic species causing leaf anthracnose of rubber tree in Hainan province of China. Seven days post treatment, the inhibition rate of *B. velezensis* 504 against *C*. *siamense* and *C*. *australisinense* were 61.85% and 66.67% respectively (**Figure 8B**). These results suggest that *B*. *velezensis* 504 can efficiently inhibit the growth of *B*. *cinerea*, *M*. *oryzae*, *C*. *siamense* and *C*. *australisinense.*

To determine whether *B*. *velezensis* 504 has the ability to promote plant growth, we formulated it into the commercially available bio-fertilizers in the form of liquid and powder-based, which were applied in the sowing time of pak choi (*Brassica campestris* sp. *chinensis* L.) in plastic greenhouse conditions. We found that the dry weight of pak choi was increased above 40% when the liquid bio-fertilizers of *B*. *velezensis* 504 was applied with the dilution of 10 or 50 times (**Figure 8C**), but no significant difference in root lengths of pak choi was observed when application of liquid and powder-based bio-fertilizers (data not shown), indicating that *B*. *velezensis* 504 is capable of stimulating plant growth. In addition, some assays relative to plant probiotic traits showed that *B*. *velezensis* 504 could secrete protease and produce siderophore (**Figure 8D**), but did not have cellulase activity and the ability to degrade inorganic phosphorus and potassium (**Supplementary Figure S5**). From these results, we conclude that *B*. *velezensis* 504 is a versatile plant probiotic bacterium.

## 4 Discussion

In this study, we mined the genomic data of *B*. *velezensis* 504, and the comparative transcriptomic data of *Xoc* RS105 treated by the CFSs of *B*. *velezensis* 504 to define DEGs, as well as analyzed the potential of *B. velezensis* 504 for biological control and growth promotion. These studies revealed the potential biocontrol mechanisms of *B*. *velezensis* against BLS, and also indicated that *B*. *velezensis* 504 is a versatile plant probiotic bacterium.

*B*. *velezensis* 504 isolated from the rhizosphere soil of spinach had a growth-promoting effect on pak choi. Some studies showed that biocontrol mechanisms of *B*. *velezensis* against pathogenic bacteria and fungi consist of the production of antimicrobial substances, induction of plant systemic resistance and promotion of plant growth (Fan et al., 2018). For examples, *B*. *velezensis* AL7 prevent wilt disease of cotton caused by *Verticillium dahliae Kleb*, a biocontrol function mainly contributed by fengycin (Liu et al., 2021a); *X. campestris* pv. *campestris* with *B*. *velezensis* FZB42 treatment occurred distorted cells that died by cell lysis (Mácha et al., 2021); inoculation of the *B*. *subtilis* LSBS2 that produce siderophore significantly promoted plant growth and enhanced plant biomass in sesame (Nithyapriya et al., 2021).Based on comparative analysis of the GO function annotation, we determined the presence of genes encoding hydrolytic enzyme activities such as glucanase, lipase, xylanase, and amylase in *B*. *velezensis* 504, and that hydrolytic enzyme activities are beneficial to improve the environmental adaptability and tolerance of microorganisms (Yadav et al., 2018). *B. velezensis* 504 produced siderophore and had extracellular protease activity, whereas genomic analysis revealed that *B. velezensis* 504 contained six genes (*entA*, *entB*, *entC*, *entE*, *dhbF*, and *menF*) related to the siderophore biosynthetic process, which mediate the interactions between microbial members and invasion of the eukaryotic hosts they colonized. Comparative genome analysis suggested that *B. velezensis* 504 was similar with SQR9 and FZB42. Genomic differences between the three strains were assumed in the numbers and sizes of hypothetical proteins, clusters encoding secondary metabolite biosynthesis, and phage portal proteins. These results implied that *B*. *velezensis* has more biocontrol potentials and probiotic mechanisms to be explored.

*B*. *velezensis* 504 showed antagonistic activity against fungal pathogens including *F*. *graminearum*, *F*. *oxysporu*, *B*. *cinerea*, *M*. *oryzae*, *C*. *siamense* and *C*. *australisinense*. Difficidin and bacilysin have been reported to be the essential antibacterial agents produced by *B. velezensis* FZB42 to control phytopathogenic fungi and bacteria, including *Xoo* and *Xoc* (Wu et al., 2015). Approximately ten percent of the total genome sequence of FZB42 is predicted to encode at least thirteen gene clusters devoted to the synthesis of active secondary metabolites that play an essential part in the prevention and control of fungal, bacterial, viral, and nematode diseases (Borriss et al., 2019). Based on the genome analysis by the antiSMASH, fourteen gene clusters of secondary metabolite with biological activity were predicted to be present in *B*. *velezensis* 504, including cyclic lipopeptides (e.g., surfactin, bacillomycin-D, fengycin, and bacillibactin, an iron-siderophore), polyketides (e.g., macrolactin, bacillaene and difficidin), dipeptide antibiotic (bacilysin), bacteriocins (plantazolicin, amylocyclicin), and volatiles (acetoin and 2, 3-butandiol). Among them, four clusters were demonstrated to involve in the synthesis of surfactin, plantazolicin, difficidin and bacilysin, which may essential for the antibacterial activity of secondary metabolites to inhibits phytopathogenic bacteria and fungi. Difficidin, fengycin, and bacilysin in secondary metabolites are also hypothesized to be critical in the inhibition of pathogens by *B*. *velezensis* YYC (Yan et al., 2022). Bacilysin, a compound with a special structure among the metabolites of *Bacillus*, is a small-molecule peptide compound, which is one of the simplest peptide antibiotics with known structure. Bacilysin is a broad-spectrum antibiotic, and has a strong inhibitory effect on fungi (Fazle Rabbee and Baek, 2020). Therefore, we speculated that diffidin, and bacilysin in *B. velezensis* 504 CFSs are the major secondary metabolites antagonizing *Xoc* and fungal pathogens. In combination with KEGG annotation analysis, it was observed that the metabolic pathways for the synthesis of 50 antibiotics such as ansamycin, penicillin, cephalosporin, and streptomycin existed in *B*. *velezensis* 504. *B*. *velezensis* 504 provides a new microbial resource for the biological control of rice, rubber tree, wheat, and grape diseases.

Based on transcriptome analysis of *Xoc* RS105 treated by the CFSs of *B*. *velezensis* 504, we found that 77% of *Xoc* RS105 CDSs were differentially expressed by the CFSs of *B*. *velezensis* 504. The most significant differences in flagellar assembly and bacterial chemotaxis of cellular processes by the KEGG pathway were identified. The KEGG pathway enrichment analysis indicated that *B. velezensis* 504 CFSs treatment possibly affect bacterial motility, LPS production, extracellular signal perception and carbon source metabolism in *Xoc* RS105. Low level expression of type I glutamate-ammonia ligase *glnA* inhibits glutamine biosynthesis, causing glycolysis to misdirect in the TCA cycle resulting in stunted cell growth (Baliga et al., 2002). Under aerobic conditions, large amounts of energy are generated through a series of oxidative phosphorylation processes following the TCA cycle, which is the hub for energy conversion of metabolites such as proteins, sugars, starch, and lipids (Madloo et al., 2021). *B*. *velezensis* 504 can secrete antagonistic metabolites to down-regulate the expression of five genes including *rpfF*, *gumD*, *ftsZ*, *rrlA* and *glmS* in *Xoc* RS105. *rpfF* is implicated in diffusible extracellular factor (DSF) formation, while *gumD* is responsible for extracellular polysaccharide (EPS) biosynthesis, both of which are required for bacterial virulence (Feng et al., 2023). *ftsZ* is associated with cell division and influences cell metabolism (Kopacz et al., 2019). *glmS* encodes glucosamine 6-phosphate synthetase that is important for the biosynthesis of peptidoglycan, a component of the bacterial cell wall (Klein and Ferre-D’Amare, 2006). Six genes encoding T3SS (*hpa1*, *hrcC*, *hpaP*, *hrpD5*, *hrpE*, and *hpa4*) were significantly down-regulated at all three-time points. Absence of *hrcC*, *hrpD5* and *hrpE*, *Xoc* RS105 would lose pathogenicity on the host rice (Li et al., 2011; Guo et al., 2012), implying that *B*. *velezensis* 504 performs a biocontrol function by significantly down-regulating the expression of virulence factor T3SS.

More down-regulated genes function in flagellar assembly (ko02040), bacterial chemotaxis (ko02030) in cellular process, starch and sucrose metabolism (ko00500), and oxidative phosphorylation (ko00190) indicating that *B. velezensis* 504 CFSs suppress phenotypes associated with chemotaxis, and motility of *Xoc* RS105, both of which are related to virulence toward host colonization. Genes involved in bacterial chemotaxis (*flhA*, *flhB*, *flgC*, *flgK*, *flgL*, *fliC* genes and *cheA*, *cheB*, *cheD*) were downregulated to suppress bacterial flagellar synthesis and chemotactic motility, reducing bacterial pathogenicity (Cai et al., 2022). Down-regulated expression of nineteen genes in the starch and sucrose metabolism pathway could lead to inadequate intracellular energy supply to bacteria, thus affecting normal bacterial growth. Compared with the *Xoc* RS105 in XOM3 cultures, 20 genes of ribosome pathway in *Xoc* RS105 were upregulated by the CFSs of *B. velezensis* 504 and 16 genes were downregulated. *rpmA*-encoded ribosomal protein L27 interacts with tRNA substrates in the peptidyl transferase center (PTC) that located in the 50S ribosomal subunit. *P*. *aeruginosa* with decreased expression of the ribosomal protein manipulator *rplU*-*rpmA* exhibited a growth-deficient phenotype. Among them, the decreased expression of *tssM* (membrane subunit) affects numerous biological processes, including the biofilm formation, population growth ability, competition, and epiphytic fitness (Fei et al., 2022). Surprisingly, the expression levels of these genes were all reduced in RS105 by the CFSs of *B. velezensis* 504, including *pstA*, *pstB*, *pstC*, and *pstS* (related with phosphate ABC transporter), *gumH* (involved in EPS production), *lpxB* and *lptB* (associated to LPS synthesis and export), *rfbC* and *rfbA* (related with streptomycin synthesis). These suggested that the reduced expression of *rfbAC* (associated with the coding for O-antigen) in 504 CFSs-treated RS105 suppressed the biosynthesis of dTDP-rhamnose, which is the precursor for EPS, LPS and cell-wall polysaccharides.

In this study, *B*. *velezensis* 504 was demonstrated to possess antagonistic activity, specifically inhibiting the growth of *Xoc*, *Xoo* and fungal pathogens such as *B*. *cinerea*, *M*. *oryzae*, *C*. *siamense* and *C*. *australisinense*, therefore, further field experiments are needed to prove whether *B*. *velezensis* 504 is a microbial biocontrol agent with prospective applications. Several *B*. *velezensis* strains have been applied as biocontrol agents in agricultural production processes with the properties of promoting plant growth and induction of systemic resistance.

## 5 Conclusion

We demonstrate that *B*. *velezensis* 504 is a potential biocontrol resource for plant diseases, exhibiting strong antibacterial and antifungal activity, as well as plant growth-promoting traits. Furthermore, the transcriptome analysis shed light on mechanisms of how the biological control agent impaired the pathogen and interfered with the bacterial growth, providing a foundation for biological control in agriculture.

## Data availability statement

The datasets presented in this study can be found in online repositories. The names of the repository/repositories and accession number(s) can be found in the article/**Supplementary Material**.

## Author contributions

LZ, QZ, and MT designed the research; YX, LZ, and MT supervised the study; QZ, XF, YC, MW, YF, YY, GuC, YZ, ZZ, and KY analyzed the data and performed part of the experiments; LZ, YY, and QZ wrote the paper; GoC, MT, and YX critically revised the manuscript. All authors contributed to the article and approved the submitted version.
