# Supplementary material for: Antagonistic transcriptome profile reveals potential mechanisms of action on Xanthomonas oryzae pv. oryzicola by the cell-free supernatants of Bacillus velezensis 504, a versatile plant probiotic bacterium
Source: Front Cell Infect Microbiol. 2023 May 30;13:1175446. doi: 10.3389/fcimb.2023.1175446 (PMC10265122; doi:10.3389/fcimb.2023.1175446)
Supplement: Supplementary file 2 [file Image_1.pdf]

Supplementary Material

Antagonistic transcriptome profile reveals potential mechanisms of action on *Xanthomonas oryzae* pv. *oryzicola* by the cell-free supernatant of *Bacillus velezensis* 504, a versatile plant probiotic bacterium

Qi Zhou[1†], Min Tu[2†], Xue Fu[2,5], Ying Chen[1], Muyuan Wang[1], Yuan Fang[1], Yichao Yan[1], Guanyun Cheng[1], Yikun Zhang[1], Zhongfeng Zhu[1], Ke Yin[1], Youlun Xiao[4*], Lifang Zou[1,3*], Gongyou Chen[1,3]

[1] Shanghai Collaborative Innovation Center of Agri-Seeds/School of Agriculture and Biology, Shanghai Jiao Tong University, Shanghai 200240, China

[2]Rubber Research Institute, Chinese Academy of Tropical Agricultural Sciences, haikou 571101, China

[3] Key Laboratory of Microbial Metabolism, School of Life Sciences & Biotechnology, Shanghai Jiao Tong University, Shanghai 200240, China

[4]Institute of Plant Protection, Hunan Academy of Agricultural Sciences, Changsha 410125, China

[5]College of Plant Protection, Yunnan Agricultural University, Kunming 650201, China

*** Correspondence:**
Lifang Zou,

Email: zoulifang202018@sjtu.edu.cn;

Youlun Xiao,

Email: xiaoylun2003@aliyun.com

[†]These authors have contributed equally to this work.

1 Supplementary Data

1.1 Supplementary Figures

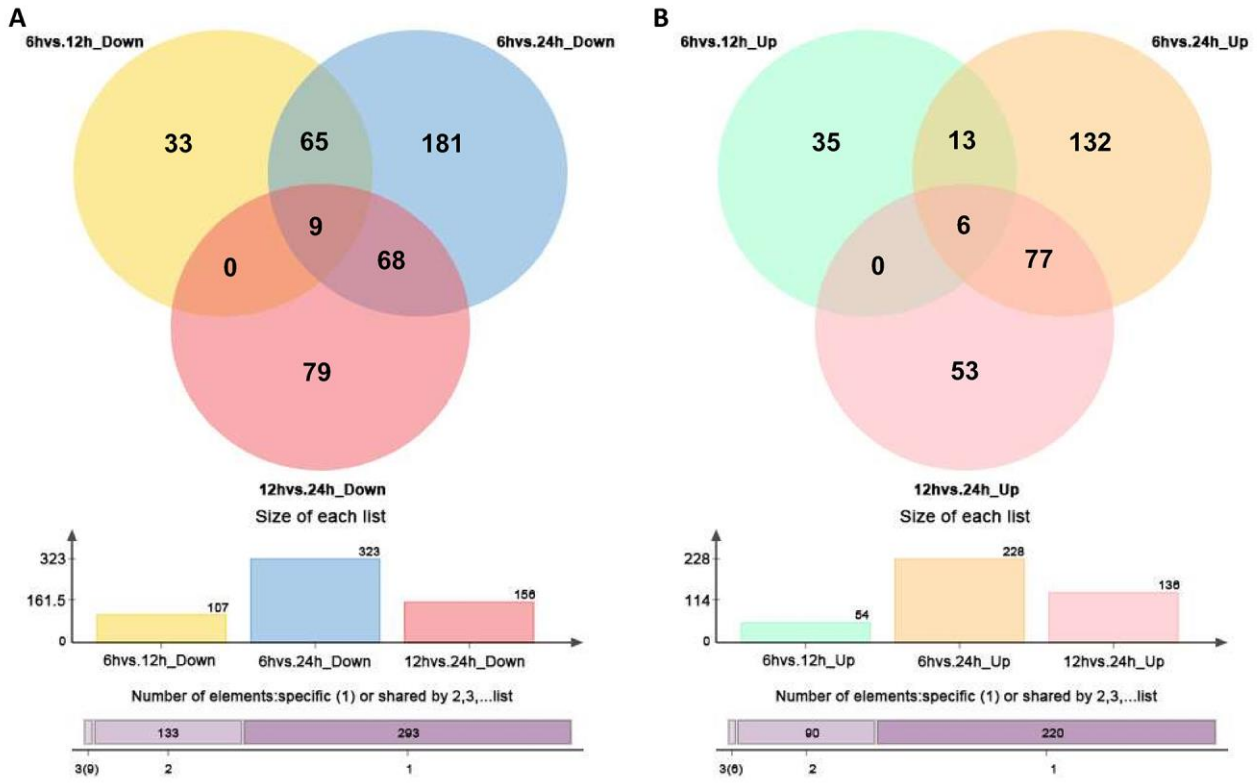

Supplementary Figure 1. Overview of transcriptome of *Xoc* RS105 under *B. velezensis* 504 CFSs conditions at different time points.

(A) Venn diagram showing the number and the overlapping down-regulated DEGs for distinct groups. (B) Venn diagram showing the number and the overlapping up-regulated DEGs for distinct groups.

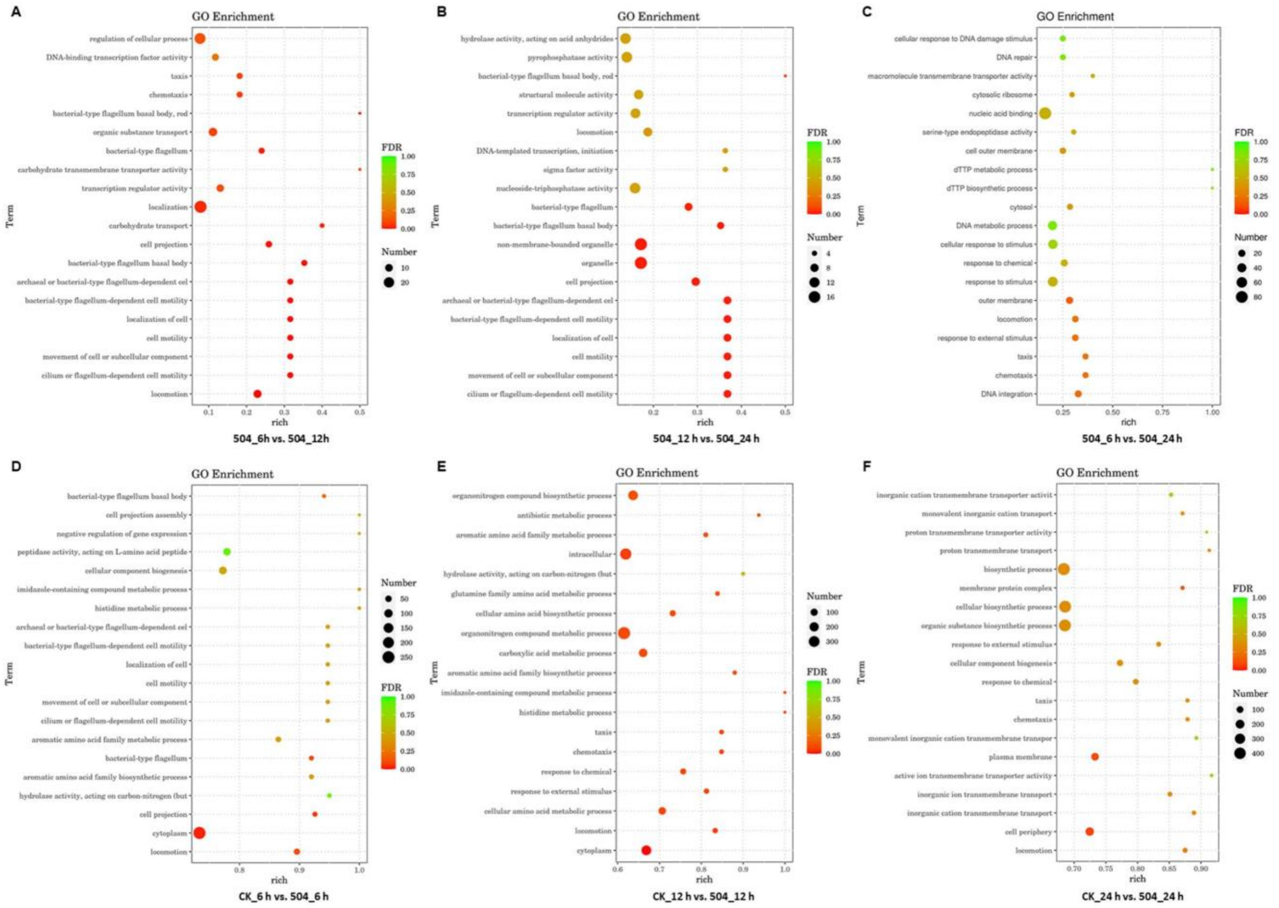

Supplementary Figure 2. Bubble diagram of top 20 ranked GO terms of DEGs.

(A) Bubble diagram showing the top 20 GO terms of DEGs between the group that 504-treated RS105 at 6 hr and 12 hr. (B) Bubble diagram showing the top 20 GO terms of DEGs between the group that 504-treated RS105 at 12hr and 24hr. (C) Bubble diagram showing the top 20 GO terms of DEGs between the group that 504-treated RS105 at 6 hr and 12 hr. The vertical axis represents GO terms, while the horizontal axis denotes the Rich factor. The magnitude of the dots represents the number of genes in the pathway, whereas their color corresponds to various Padjust ranges.

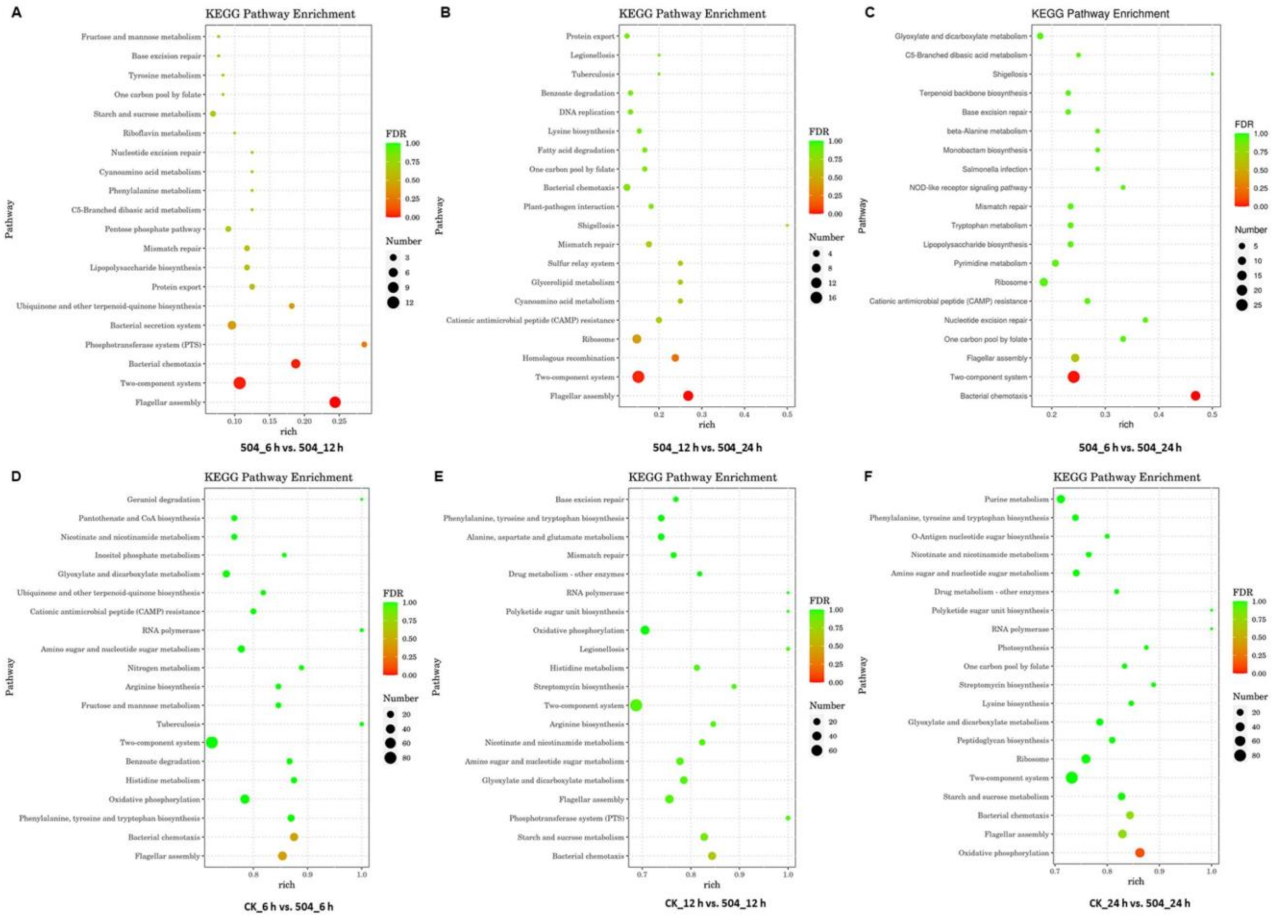

Supplementary Figure 3. Bubble diagram of top 20 ranked KEGG pathways of DEGs.

(A) Bubble diagram showing the top 20 ranked KEGG pathways of DEGs between the group that 504-treated RS105 at 6 hr and 12 hr. (B) Bubble diagram showing the top 20 ranked KEGG pathways of DEGs between the group that 504-treated RS105 at 12 hr and 24hr. (C) Bubble diagram showing the top 20 ranked KEGG pathways of DEGs between the group that 504-treated RS105 at 6 hr and 12hr. The vertical axis represents GO terms, while the horizontal axis denotes the Rich factor. The magnitude of the dots represents the number of genes in the pathway, whereas their color corresponds to various Padjust ranges.

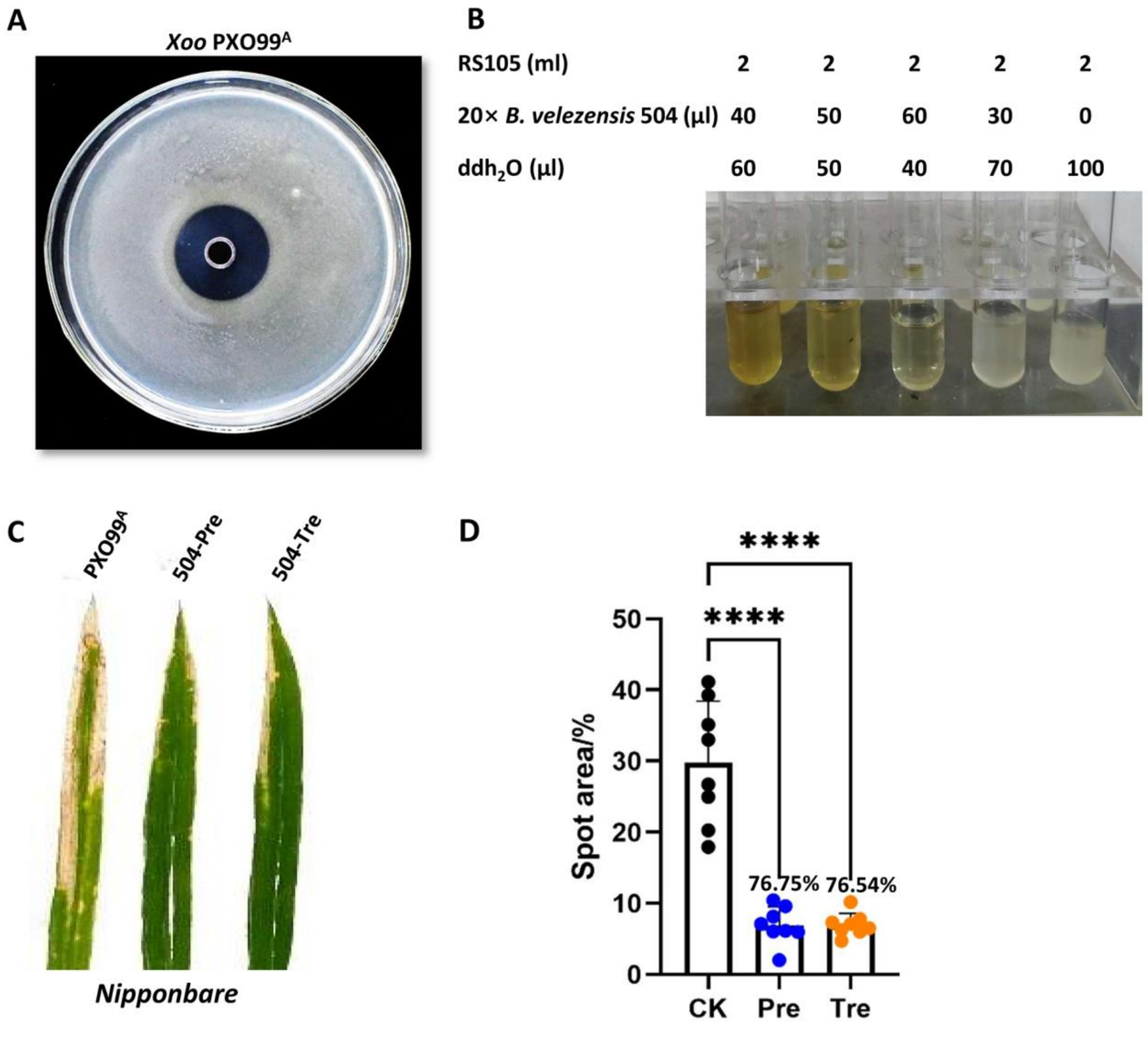

Supplementary Figure 4. Analysis of *B. velezensis* 504 CFSs antagonistic activities against *Xoo* PXO99^{A}.

(A) The CFSs of *B. velezensis* 504 can effectively inhibit the growth of *Xoo* PXO99^{A}. Three independent biological experiments were performed with similar results. (B) The assays of 50 μL 20-fold concentrated cell-free supernatants (CFSs) of *B. velezensis* 504 inhibiting against *Xoc* RS105. 30, 40, 50, and 60 μL *B. velezensis* 504 CFSs (20 times concentration) was added with 2 ml RS105 inoculum (adjusted OD_{600} to 1.0) and incubated shaking at 28 ℃ for overnight to determine the effective bactericidal concentration of *B. velezensis* 504. (C) Efficacy of *B. velezensis* 504 for control of *Xoo* PXO99^{A} in the field using the susceptible cultivars Nipponbare. The prevention (Pre) and treatment (Tre) strategies were executed as follows: *Xoo* PXO99^{A} only (Control), rice leaves sprayed with *B. velezensis* 504 12 h before inoculation with *Xoo* PXO99^{A} suspension (504-Pre), and 12 h after inoculation with *Xoo* PXO99^{A} suspension (504-Tre). (D) The bacterial blight disease of rice severity was investigated after 15 days. Data points represent means ± SD (n=8 independent leaves). The significant differences at ****$P < 0.0001$.

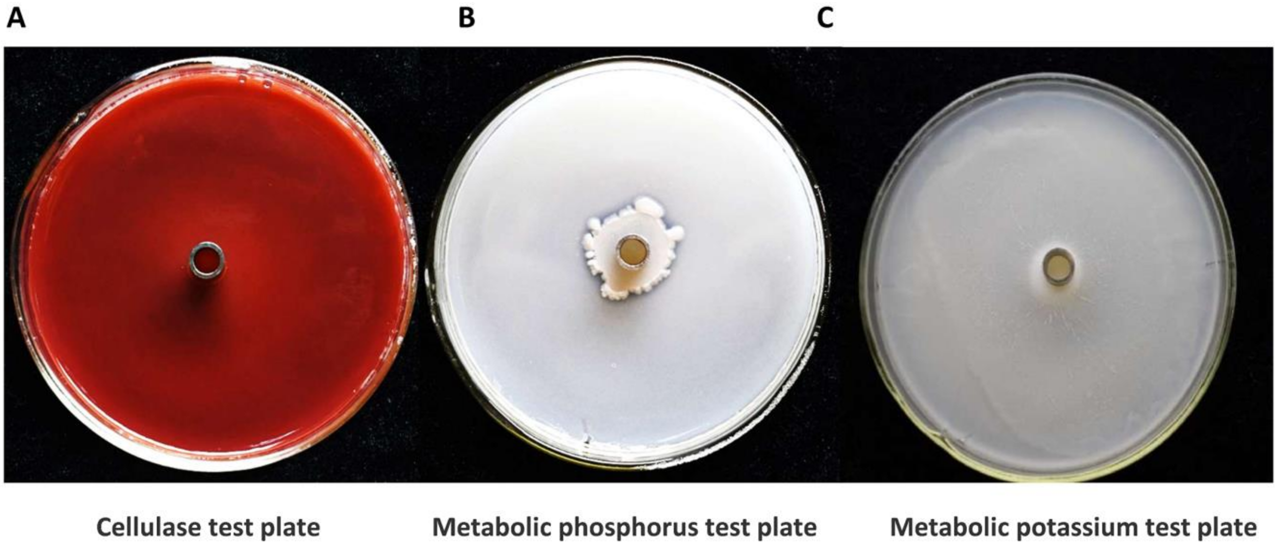

Supplementary Figure 5. Assays of *B. velezensis* 504 for cellulase production, metabolizing phosphorus and potassium.

(A) Determination of *B. velezensis* 504 cellulase activity. (B) Determination of the ability of *B. velezensis* 504 to solubilize phosphorus. (C) Determination of the ability of *B. velezensis* 504 to solubilize potassium chitinase.
